# Efficacy and safety of a therapeutic humanized FSH-blocking antibody in obesity and Alzheimer’s disease models

**DOI:** 10.1172/JCI182702

**Published:** 2025-07-15

**Authors:** Anusha R. Pallapati, Funda Korkmaz, Satish Rojekar, Steven Sims, Anurag Misra, Judit Gimenez-Roig, Aishwarya Gangadhar, Victoria Laurencin, Anisa Gumerova, Uliana Cheliadinova, Farhath Sultana, Darya Vasilyeva, Liam Cullen, Jonathan Schuermann, Jazz Munitz, Hasni Kannangara, Surabhi Parte, Georgii Pevnev, Guzel Burganova, Zehra Tumoglu, Ronit Witztum, Soleil Wizman, Natan Kramskiy, Liah Igel, Fazilet Sen, Anna Ranzenigo, Anne Macdonald, Susan Hutchison, Abraham J.P. Teunissen, Heather Burkart, Mansi Saxena, Yelena Ginsburg, Ki Goosens, Weibin Zhou, Vitaly Ryu, Ofer Moldavski, Orly Barak, Michael Pazianas, John Caminis, Shalender Bhasin, Richard Fitzgerald, Se-Min Kim, Matthew Quinn, Shozeb Haider, Susan Appt, Tal Frolinger, Clifford J. Rosen, Daria Lizneva, Yogesh K. Gupta, Tony Yuen, Mone Zaidi

**Affiliations:** 1 Mount Sinai Center for Translational Medicine and Pharmacology,; 2 Department of Pharmacological Sciences, and; 3 Department of Medicine, Icahn School of Medicine at Mount Sinai, New York, New York, USA.; 4 Greehey Children’s Cancer Research Institute and; 5 Department of Biochemistry and Structural Biology, University of Texas Health Science Center at San Antonio, San Antonio, Texas, USA.; 6 Northeastern Collaborative Access Team, Department of Chemistry and Chemical Biology, Cornell University, Ithaca, New York, USA.; 7 BioMedical Engineering and Imaging Institute,; 8 Cardiovascular Research Institute,; 9 Icahn Genomics Institute, and; 10 Department of Psychiatry, Icahn School of Medicine at Mount Sinai, New York, New York, USA.; 11 Division of Endocrinology, Diabetes and Hypertension, Brigham and Women’s Hospital, Harvard Medical School, Boston, Massachusetts, USA.; 12 Institute of Systems, Molecular and Integrative Biology, University of Liverpool, Liverpool, United Kingdom.; 13 Department of Pathology–Comparative Medicine, Wake Forest University School of Medicine, Winston-Salem, North Carolina, USA.; 14 School of Pharmacy, University College London, London, United Kingdom.; 15 MaineHealth Institute for Research, Scarborough, Maine, USA.

**Keywords:** Endocrinology, Therapeutics, Adipose tissue, Drug therapy, Neurodegeneration

## Abstract

There is growing evidence for direct actions of follicle-stimulating hormone (FSH) on tissues other than the ovaries and testes. Blocking FSH action, either genetically or pharmacologically, protects against bone loss, fat gain, and memory loss in mice. We thus developed a humanized FSH-blocking antibody, MS-Hu6, as a lead therapeutic for 3 diseases of public health magnitude — osteoporosis, obesity, and Alzheimer’s disease (AD) — that track together in postmenopausal women. Here, we report the crystal structure of MS-Hu6 and its interaction with FSH in atomistic detail. Using our Good Laboratory Practice platform (21 CFR 58), we formulated MS-Hu6 and the murine equivalent, Hf2, at an ultra-high concentration; both formulated antibodies displayed enhanced thermal and colloidal stability. A single injection of ^89^Zr-labeled MS-Hu6 revealed a β phase *t*_½_ of 79 and 132 hours for female and male mice, respectively, with retention in regions of interest. Female mice injected subcutaneously with Hf2 displayed a dose-dependent reduction in body weight and body fat, in the face of reduced free (bioavailable) FSH and unperturbed estrogen levels. Hf2 also rescued recognition memory and spatial learning loss in a context- and time-dependent manner in AD-prone *3xTg* and *APP/PS1* mice. MS-Hu6 injected into African green monkeys (8 mg/kg) intravenously, and then subcutaneously at monthly intervals, was safe, and without effects on vital signs, blood chemistries, or blood counts. There was a notable approximately 4% weight loss in all 4 monkeys after the first injection, which continued in 2 of the monkeys. We thus provide Investigational New Drug–enabling data for a planned first-in-human study.

## Abstract 2

We have developed a therapeutic humanized FSH-blocking antibody that is efficacious in reducing obesity and in preventing and treating Alzheimer’s disease.

## Graphical abstract

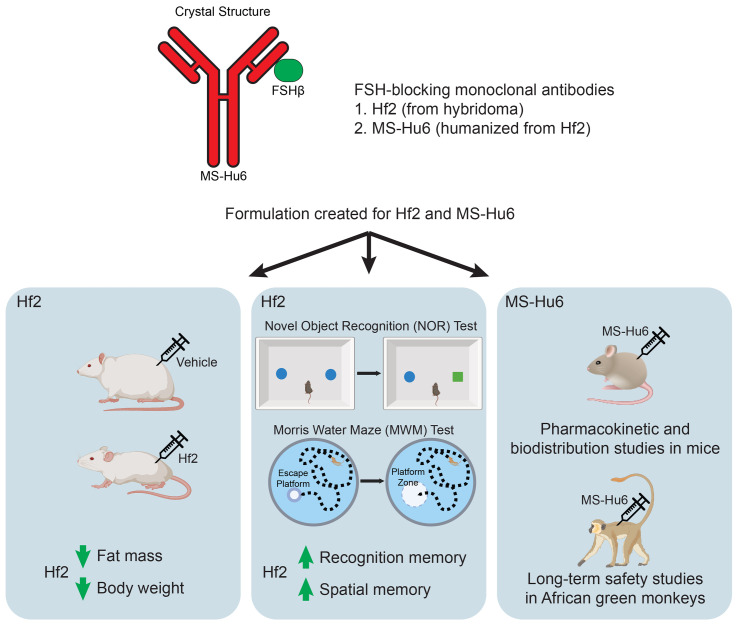

## Introduction

Studies on pituitary hormones over the past decade have transformed our understanding on how these glycoproteins work — from their historic unitary actions, mainly in the context of endocrine control, to an array of newly discovered somatic functions (reviewed in ref. 1). The first evidence for a non-traditional action of any pituitary hormone came from our demonstration that thyroid-stimulating hormone (TSH), thought solely to act on the thyroid gland, was a potent direct regulator of bone (2). It was later shown that, in contrast to TSH, which suppressed bone remodeling, follicle-stimulating hormone (FSH), hitherto considered a fertility hormone, enabled skeletal loss (3). The strongest human correlate for the latter action came from the Study of Women’s Health Across the Nation (SWAN), wherein the most rapid rates of bone loss during the perimenopausal transition occurred when serum estrogen was normal and serum FSH levels were rising (to compensate for a reduced ovarian reserve) (4, 5). Interestingly, SWAN also documented in the most rigorous manner that this normal estrogen-high FSH phase in a woman’s life also tracked with the onset of visceral adiposity and mild cognitive impairment — all of which could not conceivably be explained by low estrogen (6, 7). This led to the idea that high FSH was not only permissive to perimenopausal bone loss, but also underpinned obesity and memory loss, and could, in fact, also explain the higher lifetime risk, progression rate, and symptom burden of Alzheimer’s disease (AD) in postmenopausal women (8). Investigating this, we established that, in addition to its action in causing bone loss, FSH also acts on adipocytes to enable weight gain, and on hippocampal and cortical neurons to promote cognitive decline and AD-like pathology in mice (9, 10).

A question has thus arisen: Can we block FSH action to achieve a therapeutic benefit for osteoporosis, obesity, and AD — simultaneously — particularly in menopausal women? To test this possibility, we developed several antibodies that bind to a 13-amino-acid-long epitope of the β subunit of FSH and, by doing so, prevent its interaction with the FSH receptor (FSHR) (11–13). We found that our polyclonal FSH-blocking antibody and 2 murine monoclonal antibodies, Mf4 and Hf2, raised to the mouse and human epitopes, respectively, that differed in just 2 non-interacting amino acids, attenuated ovariectomy-induced bone loss and, in separate studies, prevented fat gain in addition to inducing beiging of white adipose tissue and thermogenesis (9, 12). The data phenocopied the effect of genetic *Fshr* haploinsufficiency, wherein mice displayed high bone mass and reduced fat content (3, 9). The data were also consistent with an interventional study in patients with prostate cancer, in which orchiectomized men displayed higher body weight and fat mass compared with men receiving the gonadotropin-releasing hormone (GnRH) agonist triptorelin — in essence, establishing that the reduction of serum FSH, even in males, can result in less weight gain (14). In separate studies, we found that the polyclonal FSH-blocking antibody attenuated the onset of the ovariectomy-induced spatial memory defect and Alzheimer’s-like neuropathology in *3xTg* mice (10).

We have since humanized Hf2 to yield 30 clones, of which we selected MS-Hu6 as the lead therapeutic candidate in silico, by using molecular dynamics to estimate global net electrostatic energy (ΔΔG), and experimentally, by measuring binding affinity (*K*_D_) by surface plasmon resonance (11). MS-Hu6 displayed the most negative ΔΔG and a *K*_D_ of 7.52 nM, approaching that of trastuzumab. The antibody bound to FSH and reduced its binding to the FSHR and expectedly inhibited both osteoclast formation and adipogenic gene expression in vitro. In parallel, in vivo studies, replicated contemporaneously in two laboratories, showed increases in bone formation and bone mass in Thermo mice (Zaidi laboratory) and in ovariectomized C57BL/6J mice (Rosen laboratory) (15). We also examined the pharmacokinetics and biodistribution of intravenous and intraperitoneal MS-Hu6 in 3 mouse models (16), C57BL/6J, CD1, and Tg32 mice (15). Finally, and importantly, we created an ultra-high-concentration (100 mg/mL) antibody formulation that displays thermal, colloidal, monomeric, structural, stress-induced, and accelerated stability (16, 17).

We now propose to move MS-Hu6 into first-in-human studies using the subcutaneous (s.c.) route, and toward this end we have developed a Good Laboratory Practice (GLP) environment consistent with *Code of Federal Regulations*, Title 21, Part 58 (21 CFR 58). Here, we present high-level Investigational New Drug–enabling (IND-enabling) preclinical data on key aspects of MS-Hu6. First, we have solved the crystal structure of MS-Hu6. Second, we provide a detailed assessment of the pharmacokinetics and biodistribution of MS-Hu6, given subcutaneously. Third, we present a comprehensive safety study in monkeys, together with preliminary weight reduction data. Fourth, we show that Hf2, the parent monoclonal antibody, binds to FSH in vivo and dose-dependently prevents body weight and fat gain in a mouse model of diet-induced obesity. Finally, and importantly, we show that Hf2 prevents the onset of memory loss in AD-prone *3xTg* and *APP/PS1* mice. The studies together not only poise us favorably to move MS-Hu6 into the clinic but also provide a compelling rationale for conducting high-quality, IND-enabling studies in academic medical centers.

## Results

### Antibody selection.

We generated 2 murine monoclonal FSH-blocking antibodies, Mf4 and Hf2, against computationally defined corresponding mouse and human FSHR-binding epitopes of FSHβ, namely LVYKDPARPNTQK and LVYKDPARPKIQK (12). In silico docking of Hf2 with FSHβ revealed that the NT and KI residues did not interact with antibody. Therefore, this relatively conserved 13-amino-acid-long epitope allowed us to test the effects of Hf2 — a murine antibody raised against the human FSHβ epitope — in murine models.

Hf2, with an IC_50_ of 6.1 nM in cell-based osteoclastogenesis assays, was thus selected for humanization (11, 12). Briefly, the variable domains of the heavy and light IgG chains, V_H_ and V_L_, were amplified from Hf2 hybridomas. A mouse-human chimeric antibody was constructed by cloning of the corresponding V_H_ and V_L_, together with human IgG_1_-C_H_ and IgK-C_L_ fragments, respectively, into pTT5 vector (11). To generate fully humanized antibodies, a bacterial expression library consisting of the antigen-binding fragments (Fab) was produced with single-site mutations introduced in the human framework flanking the complementarity-determining region (CDR), while keeping the CDR unaltered. This yielded 30 humanized Fab clones, which were tested for binding to both mouse and human FSHβ by ELISA, followed by confirmation of FSH binding by surface plasmon resonance (Biacore) and rank ordering by dissociation constants (*K*_D_). Three full-length high-affinity humanized IgGs, MS-Hu6, MS-Hu26, and MS-Hu28, were modeled with FSH and underwent in silico molecular dynamics to reveal subtle differences in binding modes and ΔΔG. MS-Hu6 was selected as the lead candidate with the most negative ΔΔG (–385 kJ/mol) and highest affinity (*K*_D_ = 7.52 nM).

Here, we report pharmacokinetic, biodistribution, and safety studies using humanized MS-Hu6. For our earlier studies in osteoporosis models, we used MS-Hu6, which elicited marked increases in bone mass (15). However, this was not the case with our obesity model, where humanized MS-Hu6 up to 100 μg, given s.c. 5 times a week, failed to reduce body weight or fat mass in mice (Supplemental Figure 1A; supplemental material available online with this article; https://doi.org/10.1172/JCI182702DS1). We therefore examined the production of murine anti-drug antibodies against MS-Hu6, which could potentially attenuate or abrogate its effect. For this, we injected male C57BL/6J mice s.c. with 250 μg MS-Hu6, Hf2, or human IgG. Mice were fed on a high-fat diet, and blood was collected every week. The presence of anti-human IgG in mouse serum was measured using an in-house ELISA, wherein human IgG (100 μg) was used as a capture antibody and mouse Fc conjugated with HRP as the detection antibody. Both MS-Hu6 and non-immune human IgG triggered clear increases in anti-human IgG production starting around week 2, and spiking at 6 weeks, whereas the murine version of MS-Hu6, Hf2, was expectedly without effect (Supplemental Figure 1B).

Before using Hf2 as a surrogate for MS-Hu6 in our long-term efficacy studies, we evaluated Hf2 and MS-Hu6 for equivalence in terms of FSH binding and FSH blocking activity. Binding to FSH was compared using the thermal shift assay. When incubated with FSH, both Hf2 and MS-Hu6 displayed a near-identical right-shift in melting temperature (*T*_m_) for the ligand-binding Fab domain (*T*_m_ ~1.8°C) (Supplemental Figure 1C). Furthermore, we studied the inhibition by Hf2 and MS-Hu6 of cAMP elevation in response to FSH (5 μg/mL) in KGN human ovarian cancer cells. There was a concentration-dependent reduction of cAMP elevation with both MS-Hu6 (IC_50_ = 0.64 nM) and Hf2 (IC_50_ = 0.51 nM) (Supplemental Figure 1D) — testifying to the equivalence of the respective antibodies in their ability to block FSH action. Furthermore, given that we were studying the effects of FSH blockade on adipocytes and neurons, we compared the EC_50_ or IC_50_, respectively, in relevant cell lines. Based on UCP1 activation in dedifferentiated brown adipocytes (Thermo cells; ref. 9), MS-Hu6 and Hf2 yielded similar EC_50_ values of 3.21 nM and 3.44 nM, respectively (Supplemental Figure 1D). Likewise, for SH-SY5Y human neuroblastoma cells (10), IC_50_ values for cAMP inhibition were 2.01 nM and 1.91 nM, respectively (Supplemental Figure 1D). The data together provide confidence that Hf2 can be used as a surrogate for MS-Hu6 in long-term efficacy studies, as has been customary for other humanized monoclonals (18–22).

### Solving the crystal structure of Hu6 and interaction with FSH.

**Figure 1:**
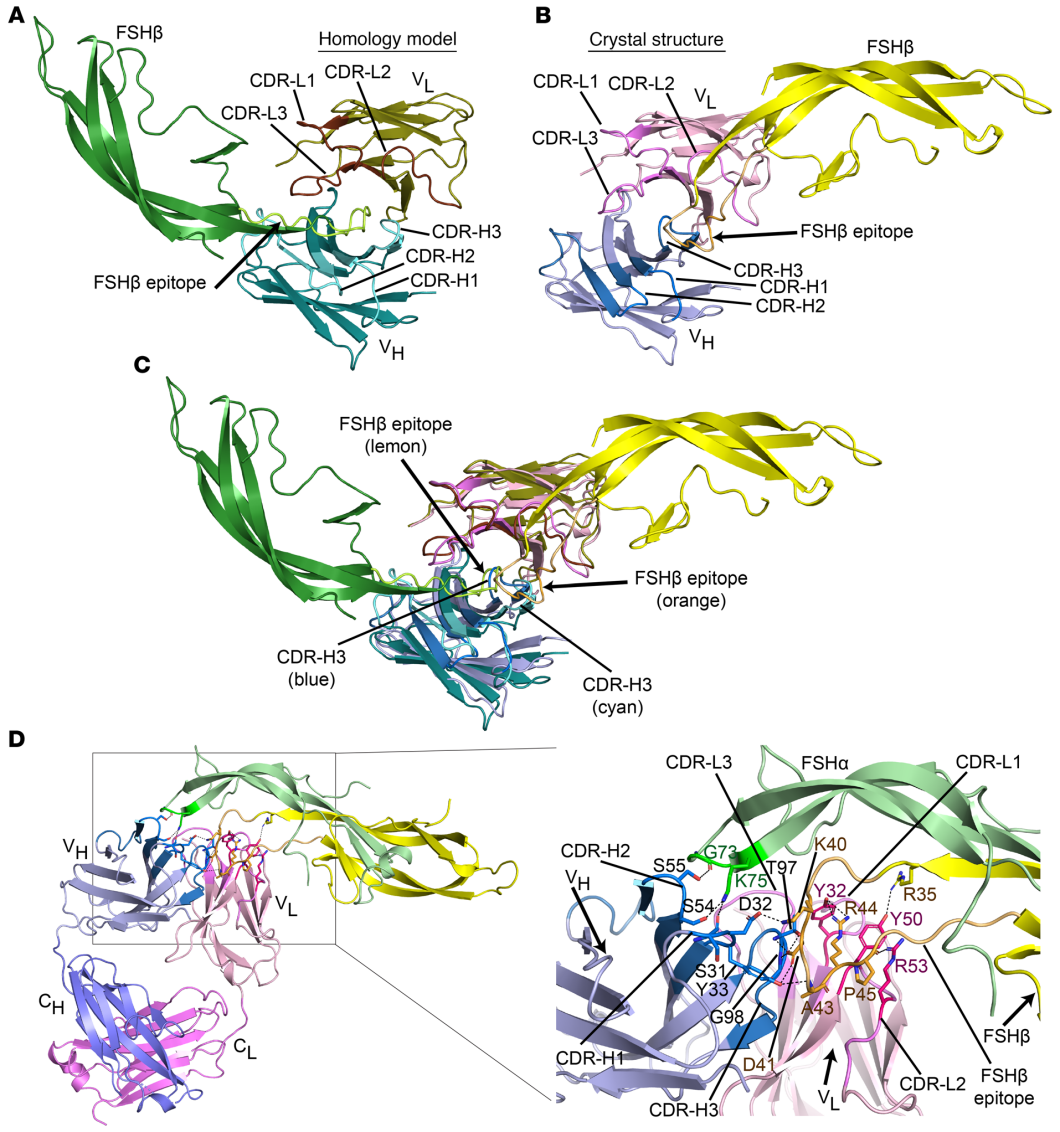
X-ray crystal structure of the Fab domain of MS-Hu6 and docked complex with FSH. (**A** and **B**) Structural comparison between the Hu6-Fab:FSHβ homology model (**A**) and the Hu6-Fab:FSHβ complex based on crystal structure of Hu6-Fab (**B**). Shown are complementarity-determining regions (CDRs), as well as the 13-mer epitope of FSHβ against which MS-Hu6 was raised. Also shown is the orientation of the FSHβ epitope with respect to the epitope-binding region of MS-Hu6, including CDR-H3. (**C**) An overlay of Fv regions of modeled and crystal-based Hu6-Fab:FSHβ complexes. (**D**) Atomistic interactions between Hu6-Fab and FSHβ. Heavy and light chains are shown in blue and pink, respectively. Variable domains of heavy chain (V_H_) and light chain (V_L_) are shown in light blue and light pink, respectively. CDRs are shown in dark blue for V_H_ (CDR-H1, CDR-H2, CDR-H3) and magenta for V_L_ (CDR-L1, CDR-L2, CDR-L3). FSHα and FSHβ chains are shown in light green and yellow, respectively. Also shown is a close-up of Hu6-Fab:FSHβ interfaces and interaction network of the 13-mer epitope (light orange) with CDRs (V_H_ and V_L_). Please refer to Supplemental Tables 1 and 2. The atomic coordinates and structure factors for Hu6-Fab have been deposited in the Protein Data Bank (PDB) with PDB ID 8VZW.

We have studied the interaction of the MS-Hu6 Fab domain with human and mouse FSHβ using molecular dynamics (11). Our homology model for human Hu6-Fab:FSH is shown in Figure 1A. However, taking a step further toward clinical development, we solved the crystal structure of the Fab fragment of MS-Hu6 (Hu6-Fab) to 2.5 Å resolution using molecular replacement (Figure 1B and Supplemental Table 1). We observed clear electron density for the light and heavy chains, except for a loop region (residues 127–130) in the heavy chain. Kabat residue numbering was applied using Abnum (www.bioinf.org.uk/abs/abnum/), while CDRs were defined by NovoPro (https://novoprolabs.com/tools/cdr) as follows: CDR-L1 (^24^RASQDISNYLS^34^), CDR-L2 (^50^YTSRLHS^56^), CDR-L3 (^89^QQGHTLPPT^97^), CDR-H1 (^31^SDYAWN^35A^), CDR-H2 (^50^SIFSSGSINYNPSLKS^65^), and CDR-H3 (^95^GGTGTDY^102^). The elbow angle at the pseudo-2-fold axes between the light and heavy chain variable and constant domains of Hu6-Fab, respectively, was derived as 134° using Elbow Angle Calculation (http://linum.proteinmodel.org/AS2TS/RBOW/index.html).

Using the Packing Angle Prediction Server (http://www.bioinf.org.uk/abs/paps/), the packing angle of V_H_ and V_L_ domains of Hu6-Fab was predicted as –46.9°, which is very close to the mean value (–45.6°) observed in 567 antibody crystal structures (23). The crystal structure of Hu6-Fab further showed that CDR-H3 moves inward to the pseudo-dyad axis and relates the packing of variable domains (V_L_/V_H_). Structural compactness arises because the inner β sheets in variable domains come closer to one end, whereas β strands from CDR-H3 (D^101^, W^103^ from V_H_) and CDR-L2 (I^44^, L^46^) are stabilized by backbone hydrogen bonding. Consistent with this, the donor (D)–acceptor (A) distances in L^46^(D)D^101^(A) [4 Å] and W^103^(D)I^44^(A) [3.9 Å] pairs are smaller than those in the equivalent pairs in other Fab structures, e.g., 4.5 and 5.4 Å in Protein Data Bank (PDB) ID 2FB4, 5.1 and 5.7 Å in PDB ID 5BMF, and >5.0 Å in PDB ID 6OGX.

Coordinates of Hu6-Fab crystal structure were used for docking with the known structure of FSH (PDB ID 1XWD) using HADDOCK 2.4 server 2 (24). The 13-amino-acid-long epitope of FSHβ against which Hu6 was raised (^37^LVYKDPARPKIQK^49^) and residues from all CDRs from V_L_ and V_H_ domains were used for efficient docking, and the lowest *z* score cluster of the complex was selected for analysis. We found that all Fv (V_L_/V_H_) CDRs, except CDR-L3, interact with FSHβ. We also observed stabilizing interactions between FSHβ and Fv. Notably, the total buried surface area (BSA) in Fv-FSH complex was estimated as 964.8 Å^2^ (using Protein Interfaces, Surfaces and Assemblies [PISA], http://www.ebi.ac.uk/pdbe/prot_int/pistart.html), which included a major contribution of 550.6 Å^2^ BSA from the FSHβ interaction whereas 414.2 Å^2^ by FSHα.

Figure 1C shows the homology model and experimental structure of Hu6-Fab together docked with FSHβ. The V_H_, V_L_, and the entire Fv regions were superposed with root mean square deviation of about 0.71 Å (96 Cα atoms) of V_L_ domains, 0.54 Å for V_H_ domains, and 1.794 Å (211 Cα atoms) for Fv (V_H_ and V_L_ together). We next compared BSAs by focusing on the Fv regions. In the crystal structure Fv, the BSA between V_H_ and V_L_ was 655.4 Å^2^ compared with a smaller BSA of 499.6 Å^2^ of the Fv homology model — suggesting that the Fv is more tightly packed in the crystal structure–based Hu6-Fab. Structural comparison of homology model–based Hu6-Fab:FSHβ and crystal Fab:FSHβ complexes further revealed that FSHβ approached the CDR differently in the two complexes (Figure 1C). This radical shift could result from the tighter packing of Fv in the crystal structure. As a result, the epitope region of FSHβ penetrated the Fv binding surface deeper in the homology model compared with the crystal-based complex. Finally, the CDR-H3 loop in crystal-based complex moved inward to the pseudo-dyad axis and appeared to modulate the interacting surface for specific interactions.

At the atomistic level, we found that residues within the 13-mer FSHβ epitope interact with CDRs of both heavy and light chains of Hu6-Fab (Figure 1D). K40 of FSHβ forms a salt bridge with D32 of CDR-H1, whereas D41 interacts with the side chain hydroxyl group of Y33 from CDR-H1. D41 also forms additional hydrogen bonds with the side chain of T97, and with backbone nitrogen atoms of T97 and G98 from CDR-H3. Backbone nitrogen of A43 in FSHβ hydrogen bonds with Y33 of CDR-H1, whereas R44 displays hydrogen bonding with Y32 from CDR-L1. Likewise, the backbone carbonyl of P45 interacts with R53 from CDR-L2, and R35, a residue proximal to the 13-mer FSHβ epitope, forms a hydrogen bond with Y50 from CDR-L2. Interestingly, in addition to specific interactions between Fv and FSHβ, FSHα also interacts with the Hu6-Fab, but only with the V_H_ domain. For example, the side chain of K75 of FSHα intrudes into the groove formed by CDR-H1 and H2 and is stabilized by electrostatic interactions with S31 and D32 from CDR-H1, and S54 from CDR-H2. An additional hydrogen bond between G73 of FSHα and side chain of S55 of CDR-H2 stabilizes the Fv-FSHβ interface.

### Tailoring of antibody formulation and assessment of thermal and monomeric stability.

We formulated both MS-Hu6 and Hf2 at an ultra-high concentration (100 mg/mL) that was deemed appropriate for future human use. Excipients approved by the US Food and Drug Administration (FDA) were used within the required range per FDA Inactive Ingredient Database. Our final optimized formulation included 20 mM phosphate buffer (pH 6.2), 260 mM sucrose, 1 mM sodium chloride, 0.001% wt/vol Tween-20, 1 mM l-methionine, and 1 mM disodium EDTA (17). During the formulation process, we employed a terminal sterilization method using a 0.22 μm sterile filter. The filtration process was performed aseptically within a clean-air environment in our GLP-compliant laboratory (Supplemental Methods), and the formulated product was filled and stored in sterile glass vials. This critical step effectively reduced aggregated particles and minimized the risk of microbial contamination.

**Figure 2:**
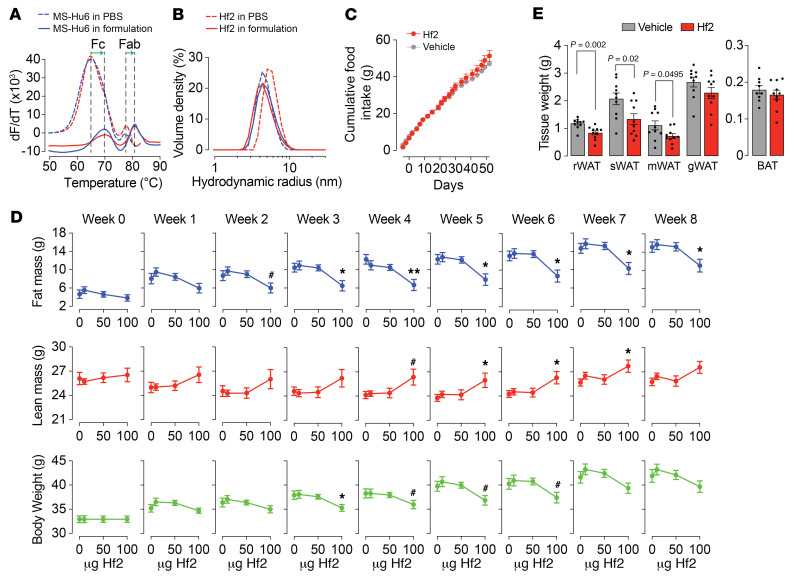
Therapeutic formulation of Hf2 prevents fat mass accrual and weight gain in mice on a high-fat diet. (**A**) Protein thermal shift assay to evaluate thermostability of MS-Hu6 and Hf2 (100 mg/mL) in therapeutic formulation versus phosphate-buffered saline (PBS). Melting curves are shown as first derivatives [(change in fluorescence)/(change in temperature)]; melting temperature (*T*_m_) is calculated from the second derivative. The notably higher *T*_m_ values for both Fc and Fab domains of both antibodies in formulation suggest greater thermostability (also see Table 1). *n* = 8 replicates. (**B**) Colloidal stability was compared using dynamic light scattering, which relies on scattering of light caused by Brownian motion of particles. Data were collected in terms of hydrodynamic radius (*R*_h_) and polydispersity index (PDI). Particle size distribution curve, *Z*-average of *R*_h_ values, and volume and size of main and other peaks are shown. *n* = 3 replicates. Formulated MS-Hu6 and Hf2 exhibited a dominant peak volume greater than 99%, with an average *R*_h_ of 4–5 nm — aligning with industry standards (<10 nm). Both formulated MS-Hu6 and Hf2 also displayed acceptable PDI values — collectively indicating the maintenance of the monomeric state (also see Table 2). (**C**–**E**) Effect of formulated Hf2 on food intake (**C**), fat mass and body weight (**D**), and tissue weights of renal white adipose tissue (rWAT), subcutaneous WAT (sWAT), mesenteric WAT (mWAT), gonadal WAT (gWAT), and brown adipose tissue (BAT) (**E**). Groups of male C57BL/6J mice, matched for body weight and fed ad libitum on a high-fat diet, were injected with formulated Hf2 at different doses (10, 50, or 100 μg/d, 5 d/wk) or formulation buffer for 8 weeks. Net food intake was measured every other day, and body weight and quantitative nuclear magnetic resonance (qNMR) measurements were made weekly. Upon sacrifice, fat depots were collected and weighed. Statistics: Two-tailed Student’s *t* test vs. vehicle; *n* = 10 mice per group; mean ± SEM; *P ≤ 0.05, **P ≤ 0.05, ^#^0.05 < *P* ≤ 0.1.

To assess thermal stability of formulated MS-Hu6 and Hf2, we used the protein thermal shift assay, as before (17). Formulated MS-Hu6 revealed higher *T*_m_ values for both Fc and Fab domains than the unformulated antibody in PBS (Δ*T*_m_ >4.5°C for Fc and >3°C for Fab, respectively) (Figure 2A and Table 1). This right-shift in *T*_m_ shows that antibodies in our tailored formulation are more stable than antibodies in PBS at equal concentrations. Furthermore, *T*_m_ values for formulated MS-Hu6 and Hf2 for their respective Fc and Fab domains were similar, indicating that the formulation imparts extreme stability to both antibodies (Figure 2A and Table 1).

We also compared the colloidal stability of MS-Hu6 and Hf2 using dynamic light scattering, a method that estimates the size (hydrodynamic radius [*R*_h_]) and homogeneity (polydispersity index [PDI]) of colloidal particles. Formulated MS-Hu6 and Hf2 exhibited a dominant peak volume greater than 99%, with an average *R*_h_ of <6 nm — aligning with industry standards (<10 nm) (Figure 2B and Table 2). While the Hf2 formulation did not show signs of aggregated peaks, there were a few minor peaks arising from antibody dimerization. Data with MS-Hu6 were essentially similar with prominent peaks at >99%. Larger particles with elevated *R*_h_ values often arise as soluble and reversible dimers, which is acceptable for biotherapeutics if they remain less than 5%. Lastly, PDI values remained less than 1, indicating that all 4 solutions were homogeneous. In all, the 100 mg/mL MS-Hu6 and Hf2 formulations displayed acceptable *R*_h_ and PDI values, indicating the maintenance of a monomeric state at ultra-high, clinically utilizable, concentrations.

### Engagement of antibody with FSH.

Given that we used Hf2 as a surrogate for in vivo efficacy studies, we evaluated its pharmacodynamic properties, or the extent to which injected Hf2 binds to its target FSH in the circulation, both in male C57BL/6J mice fed on a high-fat diet and in C57BL/6J females after ovariectomy. While total FSH was measured using a commercial FSH ELISA kit, we developed an in-house ELISA for measuring both free FSH and FSH bound to Hf2. Protein A–agarose beads precoated with anti-mouse IgG-Fc were incubated with serum collected from Hf2-injected mice. After pull-down, serum was assayed for unbound FSH, while the beads were eluted with 0.1 M glycine at pH 2.5 to measure bound FSH. Supplemental Figure 2A shows that Hf2, injected s.c. 5 days a week for 8 weeks, resulted in the binding of about 50% of serum FSH at 10 and 50 μg Hf2, whereas there was little unbound fraction left with 100 μg Hf2 (*n* = 10 mice per dose). We could not detect bound FSH with 50 and 100 μg Hf2, as the values fell below the assay’s detection limit. Thus, 100 μg was used as the Hf2 dose for studies focused on preventing obesity.

In another experiment, a group of 20 C57BL/6J female mice were ovariectomized. A week later, 10 mice were given vehicle intraperitoneally (i.p.) 5 days a week, with serum total FSH levels measured every week for 6 weeks. The other 10 mice were injected, in parallel, with Hf2 (200 μg) for the measurement of total FSH (5 mice), as well as bound and unbound FSH fractions (5 mice). Supplemental Figure 2B shows that there was a gradual increase of up to about 4-fold in total serum FSH over 4 weeks after vehicle or Hf2 treatment, with a plateau thereafter between 4 and 6 weeks. Importantly, the magnitude of serum FSH elevation at each time point was not different between vehicle- and Hf2-treated mice — illustrating that Hf2 per se did not cause a change in total serum FSH levels, as noted previously (9, 13). We also found that close to all of the circulating FSH was bound to Hf2 with very little unbound FSH available for FSHR activation, even in the presence of higher serum FSH levels (Supplemental Figure 2B). Thus, we used 200 μg Hf2 given 5 times a week for our neurobehavior experiments in ovariectomized *3xTg* mice, in which Hf2 did not lose efficacy even up to 20 weeks (see below).

As we cannot measure mouse Hf2 in mouse serum, we used MS-Hu6 as a surrogate. We injected C57BL/6J male mice s.c. with MS-Hu6, 5 days a week for 4 weeks, with serum sampling every 2 days (*n* = 5 mice). The presence of humanized MS-Hu6 in mouse serum was detected using an in-house ELISA, wherein anti–human Fab (100 μg) was used as a capture antibody and anti–human Fc conjugated with HRP as the detection antibody. We found that serum MS-Hu6 levels were detected in the 50 μg/mL range 2 days after the first injection but peaked starting at week 2 with sustained elevations above 100 μg/mL up to 4 weeks (Supplemental Figure 2C). To determine whether we could use an extended-interval dosing strategy, we used a cumulative dose of 500 μg per mouse per week and an intravenous (i.v.) protocol with serum sampling every 2 days. MS-Hu6 levels were detected at 35 μg/mL after the first injection, but rose sharply after the second injection, followed by a plateau (Supplemental Figure 2D). This suggests that an extended-interval dosing schedule using the i.v. route can indeed be used, which is the preferred route of administration for therapeutic antibodies in phase I clinical trials.

### Dose-dependent prevention of diet-induced obesity in mice.

We have shown previously that our polyclonal FSH-blocking antibody, given i.p., reduces fat accumulation in all fat depots in ovariectomized mice, mice on a high-fat diet, and mice on normal chow (9). We have also shown that a daily i.p. injection of Hf2 at 200 μg a day for 8 weeks reduces fat and body weight accrual (9). As a step toward moving these observations into people, we report here — importantly, in a GLP environment — that subcutaneous injection of Hf2, the most likely route to be used therapeutically, causes a dose-dependent reduction in body fat and body weight accrual in mice fed on a high-fat diet. For this, groups of male C57BL/6J mice, matched for body weight and fed ad libitum on a high-fat diet, were injected with *formulated* Hf2 at different doses (10, 50, or 100 μg/d, 5 d/wk) or with formulation buffer for 8 weeks. Net food intake was measured every other day, and body weight and quantitative nuclear magnetic resonance (qNMR) measurements were made weekly. Upon sacrifice, fat depots were collected and weighed.

While there was a trend toward increased food intake with Hf2 (Figure 2C), no statistically significant differences were noted up to 8 weeks of treatment. There was, however, a dose-dependent reduction in body weight and total fat mass that was significant with 100 μg Hf2, starting week 3 (Figure 2D). Manual weighing of fat depots at week 8 showed significant reductions in renal, subcutaneous, and mesenteric white adipose tissue (WAT), without changes in gonadal WAT or interscapular brown adipose tissue (Figure 2E). This therapeutic effect of Hf2 in preventing adiposity is consistent with detectable Hf2 levels in the circulation, as well as with a marked sequestration of serum FSH to yield minimal unbound (bioavailable) FSH (Supplemental Figure 2A).

We also found increases in lean mass with Hf2 beginning week 5 (Figure 2D). However, these apparent increases in lean mass in Hf2-treated mice were not due to changes in muscle or liver mass, which are the main determinants of qNMR-based lean mass measurements (Supplemental Figure 3A). There were no differences in the expression of genes associated with muscle strength (Supplemental Figure 3B). Instead, the differences in lean mass arose from reduced tissue water mass (also a determinant of the lean mass measure on qNMR) in the control group fed on a high-fat diet (Supplemental Figure 3C). Thus, when tissue water mass was subtracted from the lean mass measurement to yield the corrected measure — dry lean mass — the difference between vehicle- and Hf2-treated mice was lost (Supplemental Figure 3C). Overall, therefore, subcutaneously injected Hf2 at a minimal effective dose of 100 μg/d, for 5 days a week, sequesters nearly all circulating FSH to reduce body weight by 7% and fat mass by 38% (at week 3).

We do not expect an effect of Hf2 on serum sex steroid levels in male mice on a high-fat diet. However, given that MS-Hu6 could potentially be used in women of reproductive age, we studied the effect of Hf2 on the female reproductive system by examining the estrus cycle and uterine weight in 12-week-old female C57BL/6J mice treated with Hf2 (200 μg) or vehicle 3 days a week for 6 weeks. There was no evidence on crystal violet staining of stage-specific differences in the estrus cycle between vehicle- and Hf2-treated mice (Supplemental Figure 4A). There were also no differences in uterine weights (Supplemental Figure 4B). Serum estrogen levels measured in mice on a high-fat diet also showed no difference between vehicle- and Hf2-treated groups (Supplemental Figure 4C).

### Prevention and treatment of memory loss in AD-prone mice.

**Figure 3:**
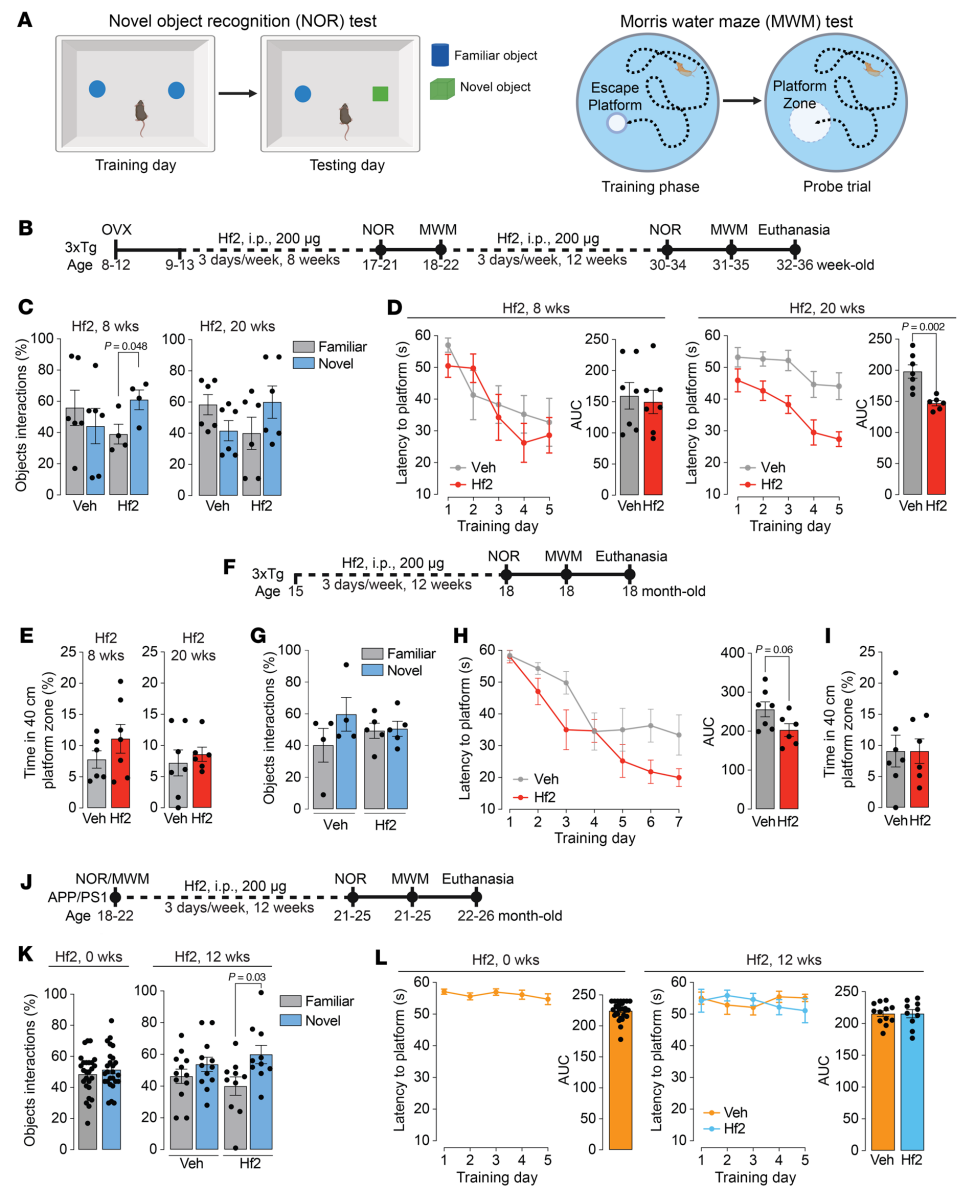
Hf2 protects against domain-specific and time-dependent memory loss in AD-prone mice. (**A**) Groups of ovariectomized or aged AD-prone mice were injected with Hf2 and subjected to the novel object recognition (NOR) test for recognition memory and the Morris water maze (MWM) test for learning and spatial memory acquisition (training phase), and retrieval of consolidated spatial memory (probe trial). (**B**) A prevention protocol involved injection of Hf2 into ovariectomized *3xTg* mice, initially for 8 weeks, and then for a further 12 weeks for a total of 20 weeks. (**C**–**E**) With Hf2, there was a significant (*P* = 0.048) increase in novel object interactions at 8 weeks, but not at 20 weeks (**C**), a significant (*P* = 0.002) reduction in latency to platform at 20 weeks, but not at 8 weeks (**D**), and no difference in spatial memory retrieval (time in the 40 cm platform zone) (probe trial) at either time point (**E**) (*n* = 7 mice per group). (**F**–**I**) In a treatment protocol, Hf2 was injected into 15-month-old *3xTg* mice for 12 weeks (**F**); this revealed no effect on object interactions in the NOR test (**G**), a marked (*P* = 0.06) reduction in latency to platform (**H**), and no effect on the probe trial in the MWM test (**I**) (*n* = 6–7 mice per group). (**J**–**L**) A complementary treatment protocol in which Hf2 was injected into 18- to 22-month-old *APP/PS1* mice for 12 weeks (**J**) revealed a significant (*P* = 0.03) increase in novel object interaction (**K**), but no difference in latency to platform (**L**) (*n* = 10–12 mice per group). Statistics: Two-tailed Student’s *t* test; mean ± SEM. AUC, area under the curve.

We have shown recently that FSH directly interacts with FSHR-positive neurons in AD-vulnerable areas in mouse brain, including the granular layer of the hippocampal gyrus and the entorhinal cortex, to trigger spatial memory loss in *3xTg* mice (10). Stereotactic injection of *Fshr* siRNA into the hippocampus prevents ovariectomy-induced memory loss, as does the systemic administration of our polyclonal FSH-blocking antibody (10) or global deletion of *Fshr* (25). We also found that the antibody prevents β-amyloid accumulation in 9-month-old aging *APP/PS1* mice, but without effects on memory (10). Overall, our data establish a direct central action of FSH on memory loss and validate FSH as a therapeutic target. Therefore, to study the effect of Hf2, as a surrogate for MS-Hu6, on memory, we chose 2 tests from our neurobehavioral testing platform: the novel object recognition test for recognition memory and the Morris water maze test for spatial memory acquisition and retrieval (Figure 3A).

For both tests, we used *3xTg* transgenic mice that overexpress 3 mutated human AD genes, *APP^K670N/M671L^*, *MAPT^P301L^*, and *PSEN1^M146V^*, and develop cognitive defects as early as about 4 months — the phenotype is known to be accelerated upon ovariectomy. We thus ovariectomized *3xTg* mice aged between 8 and 12 weeks followed, a week later, by vehicle or Hf2 injection initially for 8 weeks and extended for a further 12 weeks (i.p., 200 μg, 3 d/wk) (Figure 3B). In the novel object recognition test, mice with total object exploration of less than 5% were excluded from the analysis. There was a significant preference toward novel object in response to Hf2 after 8 weeks of injection, but not with vehicle (Figure 3C). While a trend remained, significance was lost after 12 further weeks of Hf2 (Figure 3C), suggesting an early effect of Hf2 in protecting against recognition memory loss.

The Morris water maze test consists of a training phase, which reports learning and memory acquisition, followed by a probe trial that reports the retrieval of consolidated memory (Supplemental Methods). While Hf2 failed to rescue the modest learning and acquisition memory phenotype at 8 weeks, there was a highly significant prevention after an additional 12 weeks of treatment (Figure 3D). However, spatial memory retrieval, measured by the probe trial, remained unaffected (Figure 3E).

In a second set of experiments, we employed a treatment protocol using female *3xTg* mice, which developed complete recognition memory loss at 15 months. We administered Hf2 (i.p., 200 μg, 3 d/wk) or vehicle for 12 weeks, followed by the novel object recognition and Morris water maze tests (Figure 3F). Mice with total object exploration of less than 5% were excluded from the analysis, as before. Hf2 failed to rescue recognition memory, which had been lost completely (Figure 3G). However, it rescued the more modestly impaired spatial learning and acquisition memory (Figure 3H), but without an effect on memory retrieval on the probe test (Figure 3I).

Finally, we used 18- to 22-month-old *APP/PS1* mice that harbor human *APP^K670N/M671L^* and *PSEN1^ΔE9^* mutations, without tau pathology (26), and, unlike *3xTg* mice, display a less pronounced phenotype with overt cognitive impairment at about 12 months (26–28). We administered Hf2 (i.p., 200 μg, 3 d/wk) or vehicle for 12 weeks, followed by the novel object recognition and Morris water maze tests (Figure 3J). Mice with total object exploration of less than 5% were excluded in the novel object recognition test. As expected, aged *APP/PS1* mice showed significant impairments in both novel object recognition and Morris water maze testing at baseline (Figure 3, K and L). We found that Hf2 rescued the established recognition memory impairment (Figure 3K), but not the spatial learning and acquisition defect (Figure 3L). Given that there was a complete loss of spatial learning and acquisition, we did not conduct the probe test for memory retrieval. The data collectively demonstrate clear protection of memory loss by FSH blockade in both the prevention and treatment settings; however, it is also clear that the effects of FSH blockade on recognition memory, spatial learning and acquisition, and memory retrieval are likely time- and context-dependent.

### Acute safety in African green monkeys.

**Figure 4:**
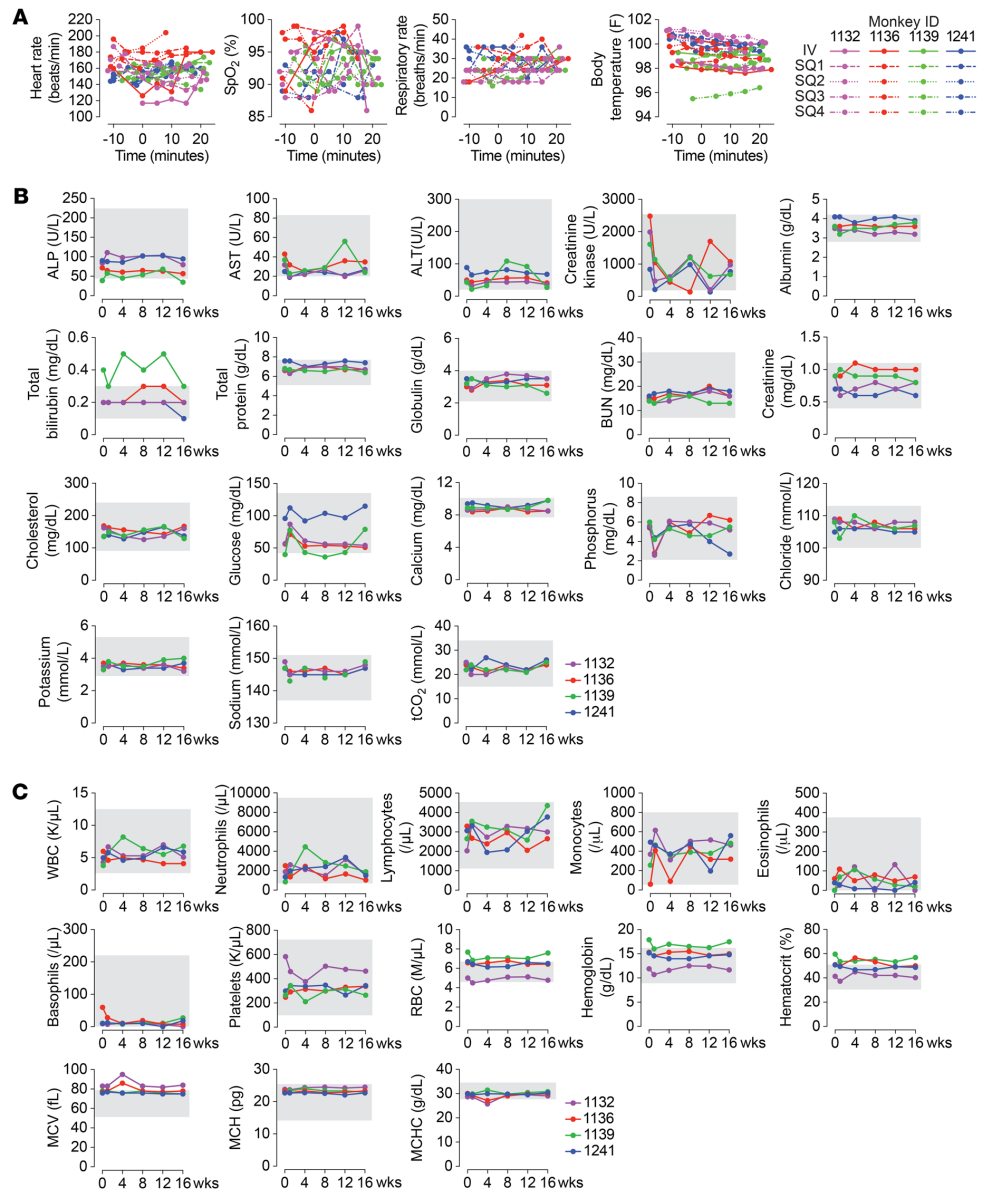
Acute and long-term safety of MS-Hu6 in African green monkeys. Retired 18- to 23-year-old female monkeys (*n* = 4; IDs: 1132, 1136, 1139, 1241) were infused i.v. with MS-Hu6 (8 mg/kg), given over 1 minute, and subsequently received 4 further s.c. injections (8 mg/kg) 30 days apart (SQ1–SQ4). Vital signs (**A**) were monitored over 20 minutes after injection, and blood chemistries (**B**) and blood cell counts (**C**) were evaluated over 16 weeks. Age-appropriate reference ranges are shown as shaded areas (35). ALP, alkaline phosphatase; ALT, alanine aminotransferase; BUN, blood urea nitrogen; MCH, mean corpuscular hemoglobin; MCHC, mean corpuscular hemoglobin concentration; MCV, mean corpuscular volume.

To study the safety of MS-Hu6 in a nonhuman primate model, we used the African green monkey (*Chlorocebus aethiops*). Four retired female monkeys (18–23 years old) were fasted overnight before sedation and anesthesia (ketamine, 10–15 mg/kg). Sedated monkeys were maintained on a heated air blanket, and 8 mL blood was collected before each MS-Hu6 injection. The monkeys were first infused i.v. with MS-Hu6 (8 mg/kg), given over 1 minute, with supplemental subcutaneous fluids (~100 mL) for hydration and nutrition. They subsequently received 4 further s.c. injections (8 mg/kg) 4 weeks apart. No acute signs of adverse reactions, including changes in skin color, heart rate, arterial oxygen saturation (SpO_2_), respiratory rate, or rectal temperature, were observed up to 20 minutes after the i.v. infusion and 4 further s.c. injections (Figure 4A). Complete serum chemistries and blood counts, including liver and kidney function tests, remained normal following each injection, with the exception of one monkey that displayed a high baseline level of serum total bilirubin (Figure 4, B and C). One monkey, 1132, displayed a body temperature lower than normal, 20 minutes after ketamine prior to the fourth s.c. MS-Hu6 injection. Body temperature normalized 20 minutes later (not shown). Reduced body temperature is not uncommon with ketamine anesthesia. Lastly, there was a small reduction in serum glucose just below the lower limit of normal in monkey 1139, but this did not result in clinical evidence of hypoglycemia.

We did, however, observe a reduction in body weight (median change, 4.06%; range 1.97%–4.61%) in all 4 animals 30 days after the single i.v. infusion; this weight loss persisted in 2 of the 4 monkeys upon subsequent s.c. injections (Supplemental Figure 5). This surprising result in monkeys (not on a high-fat diet) appears consistent with our murine studies (Figure 2, D and E); nonetheless, the absence of a control group prevents us from making conclusions. Importantly, however, this study shows clearly that a single i.v. bolus and subsequent s.c. injections of MS-Hu6 do not trigger clinical or biochemical safety signals over 4 months.

### Pharmacokinetics and biodistribution of MS-Hu6 in mice.

**Figure 5:**
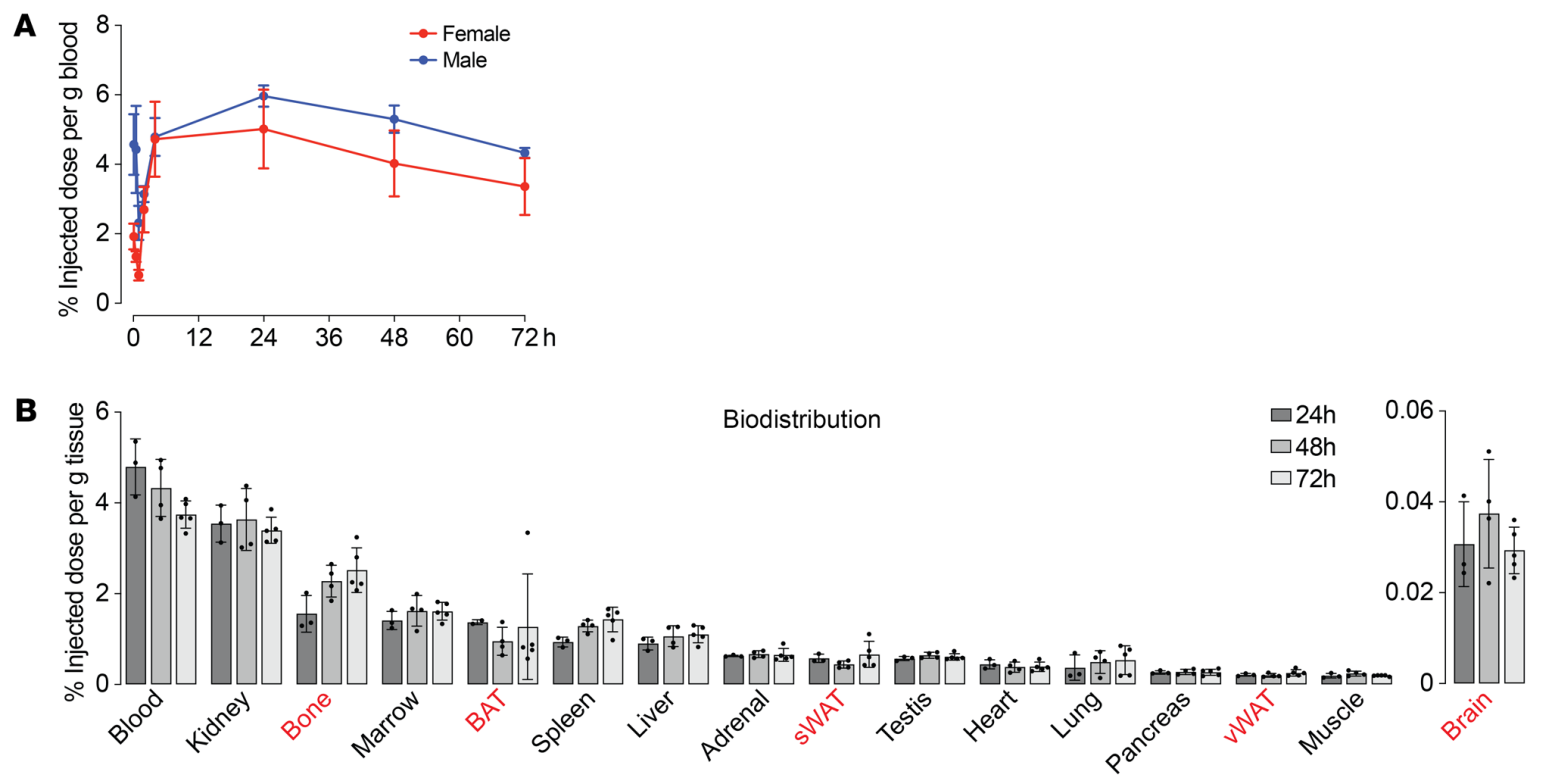
Pharmacokinetics and biodistribution of MS-Hu6 in C57BL/6J mice. (**A**) Pharmacokinetics of ^89^Zr-labeled MS-Hu6 injected as a single s.c. dose of 250 μCi into groups of male and female C57BL/6J mice (*n* = 5 mice per group). Blood counts were corrected for decay and expressed as percentage of the injected dose per gram of blood. Mean pharmacokinetic parameters calculated by non-compartmental analysis after extravascular input using PK Solver v2.0 included β phase *t*_½_, time to peak concentration (*T*_max_), peak serum concentration (*C*_max_), mean residence time (MRT), area under the curve up to 72 hours (AUC), volume of distribution (Vz/F), and apparent clearance (Cl/F) (also see Table 3). (**B**) For biodistribution studies, a single dose of ^89^Zr-MS-Hu6 (250 μCi) was injected s.c. into groups of male C57BL/6J mice (*n* = 5 mice for each time point). Blood was drawn and tissues, including bone, subcutaneous and visceral WAT (sWAT and vWAT), brown adipose tissue (BAT), brain, kidney, liver, muscle, lung, heart, spleen, testis, adrenal gland, and pancreas, were isolated at 24, 48, and 72 hours. γ-Counts were corrected for decay and expressed as percentage of the injected dose per gram of tissue or blood.

For pharmacokinetic studies, ^89^Zr-MS-Hu6 was injected s.c. (250 μCi) to groups of 3-month-old male and female C57BL/6J mice. Blood (~5 μL) was collected via the tail vein at 0, 0.5, 1, 2, 4, 24, and 72 hours, and γ-counts were corrected for decay and expressed as a percentage of the injected dose per gram of blood. Figure 5A and Table 3 show the concentration-time profile of MS-Hu6. Mean pharmacokinetic parameters, calculated by non-compartmental analysis after extravascular input (linear trapezoidal method) using PK Solver v2.0 (https://www.boomer.org/boomer/software/pksolver.zip), revealed sex differences in key pharmacokinetic parameters, including *t*_½_, area under the curve (AUC), and mean residence time (MRT) (Figure 5A and Table 3). AUC was 1.22-fold higher in male than in female mice, indicating a slight reduction in MS-Hu6 clearance in male mice. Consistent with this, the *t*_½_ and MRT of ^89^Zr-Hu6 were 1.65- and 1.66-fold higher, respectively, in male compared with female mice. Increased MRT and *t*_½_ values, together with reduced volume of distribution (Vz/F) and clearance (Cl/F), suggest that ^89^Zr-MS-Hu6 may be more persistent in male mice. Conversely, in female mice, these parameters suggest a higher elimination rate for MS-Hu6.

For biodistribution studies, a single dose of ^89^Zr-MS-Hu6 (250 μCi) was injected s.c. into groups of male C57BL/6J mice, which was followed at 24, 48, and 72 hours by terminal blood draw and harvesting of bone, fat depots, brain, kidney, liver, muscle, lung, heart, spleen, testis, adrenal gland, and pancreas. γ-Counts were corrected for decay and expressed as a percentage of the injected dose per gram of tissue or blood. ^89^Zr-MS-Hu6 distributed primarily to the kidney, bone, bone marrow, and spleen (Figure 5B). Blood levels of MS-Hu6 that were sustained at least up to 72 hours were consistent with the pharmacokinetic studies (Figure 5A and Table 3) and suggest a slow distribution to target sites over time. Substantial concentrations of ^89^Zr-MS-Hu6 were also detected in bone, bone marrow, subcutaneous white adipose tissue (WAT), visceral WAT, and brown adipose tissue (Figure 5B). Most notable, and therapeutically relevant, is that the ^89^Zr-MS-Hu6 content of bone tissue was 1.61-fold higher at the 72-hour time point compared with 24 hours, suggesting enhanced persistence over time. Antibody content in fat tissues remained constant. Minimal levels of ^89^Zr-MS-Hu6 were consistently found in isolated brain samples at all time points. This observation aligns with the well-known limited penetration of IgGs into brain tissue (0.05%–0.1%) (Figure 5B).

## Discussion

Our studies over the past two decades have unmasked new roles for pituitary hormones of physiologic and medical significance and have established high FSH as causal to bone loss, obesity, and memory loss that track together across the transition to menopause. Briefly, FSH stimulates bone loss by triggering osteoclastic bone resorption and inhibiting osteoblast formation, while inhibition of FSH signaling prevents bone loss (3, 12, 13). We have also shown that FSH acts on FSHRs on adipocytes to inhibit UCP1 activation, and that blockade of this action either genetically in *Fshr^+/–^* mice or pharmacologically by our FSH-blocking antibody reduces body fat by stimulating UCP1 activation, mitochondrial biogenesis, and energy expenditure (9). More recently, we discovered that FSH acts on neuronal FSHRs, mainly localized to AD-vulnerable brain regions, to enhance the expression of C/EBPβ. The latter stimulates the expression of arginine endopeptidase, a δ-secretase, which cleaves APP to small peptides that aggregate to form β-amyloid plaques, as well as tau to yield neurofibrillary tangles. The resulting spatial memory loss in AD-prone *3xTg* mice is prevented by siRNA knockdown of the hippocampal *Fshr*, in compound *3xTg* *Fshr^–/–^* mutants, and by use of our polyclonal FSH-blocking antibody (10, 25). These initial proof-of-concept studies have paved the way for treatment of 3 disorders of public health magnitude — osteoporosis, obesity, and AD — simultaneously and with a single FSH inhibitor (3, 6, 7, 9, 10, 12, 29). We thus developed and characterized a lead therapeutic FSH-blocking antibody, MS-Hu6 (11).

We have solved the crystal structure of the Fab fragment of MS-Hu6, which expectedly displayed a number of similarities, including the packing angle of the V_H_ and V_L_ domains, when compared with 567 antibody crystal structures (23). However, there were certain differences, for example, in the hydrogen bond donor-acceptor distances between CDR-H3 and CDR-L2, which, at 4.0 and 3.9 Å, were smaller than the equivalent pairs in other Fab structures, such as PDB IDs 2FB4, 5BMF, and 6OGX. Furthermore, the inner β sheets in the variable domains were closer to one end, whereas β strands from CDR-H3 and CDR-L2 were stabilized by hydrogen bonding; this led to the structural compactness of MS-Hu6. We also found that, since CDR-H3 conformations were diverse and unclassifiable, additional weak antiparallel interactions of Hu6-Fab contributed to the CDR-H3 conformation that, we posit, enables tight binding to FSH. Docking with FSH further revealed that all CDRs, except CDR-L3, interacted with FSHβ, with stabilizing interactions through salt bridges and hydrogen bonding; these included amino acids, such as R35, that were part of the 13-amino-acid-long epitope to which MS-Hu6 was raised (12). Interestingly, Hu6-Fab also interacted with FSHα through the V_H_ domain via electrostatic interactions. In all, our data provide compelling evidence for target (FSHβ) capture by our lead therapeutic, MS-Hu6, as well as the structural framework for future chemical modifications that may further enhance binding affinity.

Transitioning of the antibody into people is based on certain early-obtainable requisites, one of which is an injectable formulation that displays thermal, structural, colloidal, monomeric, and, most importantly, stress-induced and accelerated stability. We generated a formulation in-house that contains an ultra-high concentration of MS-Hu6 at 100 mg/mL. This means that up to 150 mg MS-Hu6 can be injected subcutaneously in a volume of 1.5 mL, which is the upper limit of an allowable subcutaneous injection volume. We have shown previously that MS-Hu6 is stable in all respects, with acceptable clarity and viscosity (17). Here, we used the protein thermal shift assay and dynamic light scattering to not only confirm thermal and monomeric stability of MS-Hu6, but also establish that the parent antibody, Hf2, is equally stable. It is necessary that we use Hf2 for long-term efficacy studies as anti-human IgG antibodies were found to develop when humanized MS-Hu6 was injected into mice. Substitution to the murine molecule is commonplace when performing preclinical mouse studies with humanized or human antibodies (18–22).

Another issue relates to the route of administration. Prior efficacy data were from intraperitoneal injections (9, 12). However, given that subcutaneous injection is the likely route for its clinical use, we performed a full pharmacokinetic and biodistribution study in C57BL/6J mice using subcutaneous MS-Hu6. There was an unexplained difference in β phase *t*_½_ for ^89^Zr-MS-Hu6 between male and female mice, with faster elimination in females. Furthermore, using the intraperitoneal route, we found previously that, compared with C56BL/6 mice, the β phase *t*_½_ for ^89^Zr-MS-Hu6 is about 3-fold higher (expectedly) in Tg32 mice (30), in which the FcRn receptor is humanized (29 vs. 97 hours) (15). Using the same fold difference, we posit that, for the subcutaneous route, β phase *t*_½_ of 79 hours (3.4 days) and 132 hours (5.5 days), respectively, for male and female C57BL/6J mice, will translate into 10 and 15 days in Tg32 mice. This estimate is consistent with the direct prediction of *t*_½_ in humans by application of the scaling-based single-exponent method to our C57BL/6J data (31).

^89^Zr-MS-Hu6 distributed to tissues of interest, namely bone, bone marrow, and adipose tissue depots. Most notably, there was greater retention of MS-Hu6 in bone tissue at 72 hours. Albeit for unclear reasons, selective bone accumulation may account for the pro-anabolic actions of MS-Hu6 on the skeleton (15), despite circulating anti-drug antibodies shown here. Second, there was modest antibody penetration into the brain, as would be expected with IgGs (32), which utilize either uncontrolled non-specific routes or endogenous transport systems, including receptor-mediated transcytosis or carrier-mediated transport (33). The precise mechanism of MS-Hu6 transport into the brain is unclear. However, we posit that while central effects may play a role, the prevention of memory loss might also result from the reduction of bioavailable FSH at brain sites due to its binding with antibody in the periphery, as we show here (10).

Our previous study in *Cynomolgus* monkeys used ^89^Zr-MS-Hu6 (1.3 mg/kg, intravenous) for biodistribution studies and noted no adverse events (15). Here, we purposefully studied acute and chronic safety in aged, retired female African green monkeys by injecting MS-Hu6 at a high dose of 8 mg/kg via both intravenous and subcutaneous routes. There were no acute signs of adverse reactions, with normal vital signs up to 20 minutes after intravenous infusion or 4 further subcutaneous injections. Complete blood counts and serum chemistries remained largely normal following each injection. There was, however, a surprising approximately 4% reduction in body weight in all 4 monkeys 30 days after the intravenous infusion, with the weight loss persisting in 2 of the 4 monkeys that were subsequently injected subcutaneously. While this impressive weight loss is consistent with our murine studies, the discovery was serendipitous and lacks a control group.

However, our study in mice showed a clear dose-dependent reduction in body fat and body weight, of 7% and 38%, respectively. Statistical significance was obtained with either parameter at 3 weeks of subcutaneous injection of the parent murine antibody Hf2 (4 mg/kg) given 5 days a week. This provides us with a minimally effective subcutaneous dose at which serum antibody levels are maintained at about 50 μg/mL, and bioavailable (unbound) FSH is markedly reduced. Nonetheless, a question remains, given the predicted *t*_½_ in humans of about 2 weeks, of whether we can extend the interval between doses. This appears possible given data from a prior study, wherein ovariectomy-induced bone loss was prevented equally with our polyclonal antibody used at 100 μg/mouse daily or 400 μg/mouse once every 4 days (13). In this context, intravenous injections of MS-Hu6 every week maintain circulating antibody levels above 50 μg/mL — a level that we find is therapeutic.

We also found that the lean mass measure on qNMR (which also captures tissue water) was increased in Hf2-treated mice. However, this increase resulted from less tissue water in the vehicle-treated group, consistent with the known effect of a high-fat diet in reducing water intake (34). In line with this, there were no differences between the two groups in other determinants of the lean mass parameter, notably muscle or liver weight, or in gene-based indicators of muscle strength. These bulk studies nonetheless do not rule out effects of FSH blockade on muscle.

Lastly, in line with our prior data that establish a direct central action of FSH on memory loss (10), FSH blockade by Hf2 given for 8 weeks prevented ovariectomy-induced recognition memory deficit in AD-prone *3xTg* mice. However, with the progression of disease over a further 12 weeks, this protection was lost. Furthermore, Hf2 failed to reverse the severe impairment of recognition memory (novel object recognition test) and retrieval of spatial memory (probe trial in Morris water maze test) in 15-month-old *3xTg* mice. In contrast, Hf2 prevented the loss of spatial learning and memory acquisition (training phase in Morris water maze test), which had become impaired after a further 12 weeks of injection. Likewise, the impaired spatial learning and acquisition in 15-month-old mice were rescued by Hf2. Interestingly, *APP/PS1* mice displayed a different sequence in the loss of memory functions, wherein spatial learning and acquisition memory declined severely, preceding the impairment of recognition memory. Thus, in contrast to *3xTg* mice, Hf2 rescued recognition memory but not spatial learning and acquisition in *APP/PS1* mice. Together, the data suggest that FSH blockade in vivo can be of potential use in preventing and treating different domains of memory loss over different time scales, as is evident in 2 distinct mouse models prone to AD.

In summary, with the aim of moving MS-Hu6 into the clinic, we have solved its structure by x-ray crystallography; demonstrated its ability to bind to and block the action of FSH; documented antibody stability at an ultra-high concentration in a therapeutic formulation; determined its pharmacokinetics and biodistribution; established acute and chronic safety in aged African green monkeys; and, importantly, proven efficacy in reducing body weight and body fat, and in preventing and treating memory loss in mice. All of this was achieved in an in-house GLP laboratory compliant with the *Code of Federal Regulations*, Title 21, Part 58 — which enables our data to be used in an Investigational New Drug application.

## Methods

Please see Supplemental Methods for further information.

### Sex as a biological variable.

Our study examined male and female animals, and similar findings are reported for both sexes.

### Statistics.

Data were analyzed using GraphPad Prism v10 and are presented as mean ± SEM. Data distribution was first checked for normality, and pairwise comparisons were performed by unpaired 2-tailed Student’s *t* test. Statistical significance was defined as *P* less than 0.05.

### Study approval.

The Institutional Animal Care and Use Committees at Icahn School of Medicine at Mount Sinai and Wake Forest University School of Medicine evaluated and approved all mouse and monkey experiments and procedures.

### Data availability.

All data in the article and supplemental material are available in the Supporting Data Values file.

## Author contributions

ARP, FK, SR, SS, A Misra, JGR, A Gangadhar, VL, A Gumerova, UC, F Sultana, DV, LC, JS, JM, SP, GP, ZT, AR, and HB performed experimentation, data acquisition, and data analyses. GB, RW, SW, NK, LI, F Sen, and AJPT performed data curation and formal analysis. S Hutchison, OM, and OB provided data management and provenance. A Macdonald, MS, OB, MP, and JC performed GLP management and developed methodology. HK, SMK, MQ, TF, and DL performed data curation, quality control, and reanalysis. YG, KG, WZ, VR, SB, RF, S Haider, SA, CJR, DL, and YKG conceptualized the study and developed methodology. TY and MZ conceptualized and supervised the study, wrote the original draft of the manuscript, and reviewed and edited the manuscript. The order of co–first authors was designated based on the amount of time each author contributed to the study.

## Supplementary Material

Supplemental data

Supporting data values

## Figures and Tables

**Table 2:**
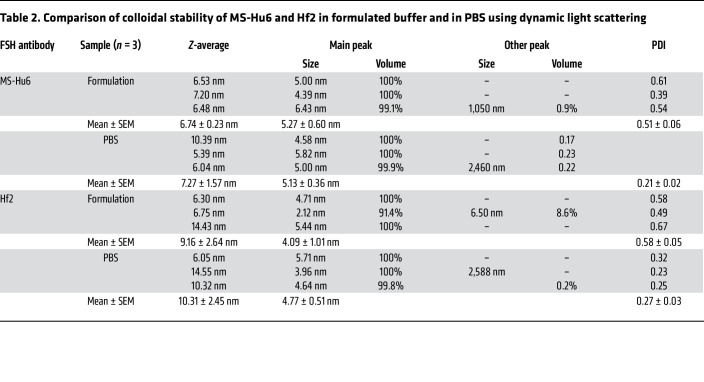
Comparison of colloidal stability of MS-Hu6 and Hf2 in formulated buffer and in PBS using dynamic light scattering

**Table 1:**
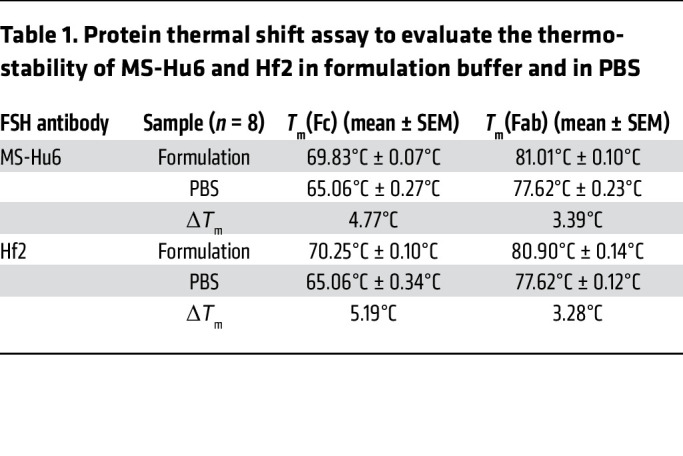
Protein thermal shift assay to evaluate the thermostability of MS-Hu6 and Hf2 in formulation buffer and in PBS

**Table 3:**
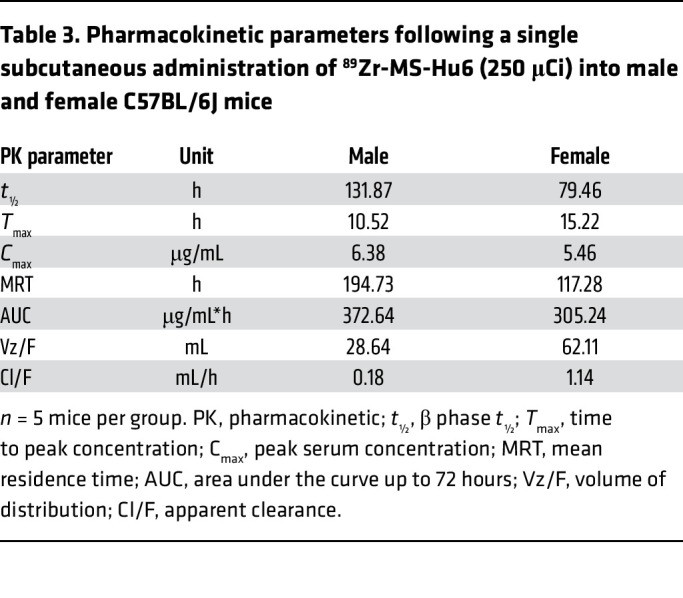
Pharmacokinetic parameters following a single subcutaneous administration of ^89^Zr-MS-Hu6 (250 μCi) into male and female C57BL/6J mice

## Version 1 07/15/2025

In-Press Preview

## Version 2 09/02/2025

Electronic publication
