# Supplementary material for: Efficacy and safety of a therapeutic humanized FSH-blocking antibody in obesity and Alzheimer’s disease models
Source: J Clin Invest. 2025 Jul 15;135(17):e182702. doi: 10.1172/JCI182702 (PMC12404759; doi:10.1172/JCI182702)
Supplement: Supplemental data [file jci-135-182702-s238.pdf]

SUPPLEMENTAL MATERIAL

SUPPLEMENTAL METHODS

Mice

C57BL/6J, *3xTg* and *APP/PS1* mice were obtained from Jackson Laboratory (stock# 000664, 034830 and 034832 respectively). All mice were housed at a temperature of 22 °C with a 12-h/12-h light/dark cycle while being kept in a pathogen–free environment. Water and food were available as needed. The tests were carried out in accordance with animal care guidelines of the Icahn School of Medicine at Mount Sinai and the NIH. The Institutional Animal Care and Use Committee (IACUC) at the Icahn School of Medicine at Mount Sinai evaluated and approved the procedure.

Anti–Drug Antibodies

Anti–drug antibodies (ADA) lead to reduced availability and lower the effective concentration of drug due to neutralization. To test whether our FSH–blocking antibody MS-Hu6 triggers ADA formation in mice, we injected male C57BL/6J mice subcutaneously with 250 μg humanized MS-Hu6, murine Hf2 or human IgG. Mice were fed on a high–fat diet with blood collected every week. Levels of ADAs (anti–human IgGs) in mouse serum were measured using an in–house ELISA, in which human IgG (100 μg, Sigma, Catalog #I4506), immobilized to the plate, was used to capture the ADAs, and mouse Fc–conjugated with HRP (ThermoFisher Scientific, Catalog #A16084) was used to detect the complex.

Protein Thermal Shift Assay

The protein thermal shift assay used a fluorescent reporter, Sypro–Orange (Applied Biosystems, Catalog #4461146), to detect hydrophobic domains that are exposed following the

heat–induced unfolding of globular proteins. To determine whether FSH binds to MS-Hu6 and Hf2, we incubated 1 μg/μL, each, in PBS with or without human FSH (0.5 μg/μL) at room temperature for 30 minutes. Fluorescence was captured sequentially at 0.3 °C increments using a StepOne Plus Thermocycler (Applied Biosystems). For the assessment of the stability of MS-Hu6 and Hf2 in the ultra–high formulation and PBS, we used 100 mg/mL antibodies. Melting temperature, Tm, was calculated based on the inflection point of the melt curve.

Cyclic AMP Assay

A cAMP assay was used to determine the ability of MS-Hu6 and Hf2 to block the action of FSH in KGN cells (Creative Bioarray, Catalog #CSC-C9202W), a human ovarian granulosa cell line, as well as in neuroblastoma cells (SHS-Y5Y, ATCC, Catalog #CRL-2266)—both of which express the FSHR (1). MS–Hu6 and Hf2 were diluted to obtain concentrations from 0.02 to 20 nM for KGN cells and from 0.2 to 25 nM for SH-SY5Y cells; each concentration was incubated with 5 μg/mL human FSH (GoldenWest, Catalog #TLIH-A1110) at 4 °C for 1 hour. The mixture was then used to treat KGN or SH-SY5Y cells for 20 and 60 minutes, respectively. cAMP levels were measured in cell lysates using an ELISA (Cayman Chemical Cyclic AMP Kit, #581001). IC_{50} values were determined.

Thermo Cell Differentiation and Luciferase Assay

To determine the EC_{50} for the FSH–blocking activity of Hf2 and MS-Hu6 on adipocytes, dedifferentiated brown adipocytes (Thermo cells) (2) were used for the measurement of UCP1–driven LUC activity. Briefly, cells were cultured in Dulbecco's Modified Eagle's Medium supplemented with 10% FBS and 1% penicillin/streptomycin. Upon 100% confluence (day 0), differentiation was induced with medium containing 10% FBS, 5 μg/mL insulin, 1 nM T_3, 0.125 mM indomethacin, 2 μg/mL dexamethasone, and 0.125 mM 3-isobutyl-1-methylxanthine for 2

days. From day 2 on, cells were cultured only in the presence of insulin and T_3 for 8 days. Cells were then induced with β3 agonist (CL316243, 10^{-7} M, Tocris, Catalog #1499) and pre–incubated with FSH (30 ng/ml) and different concentrations of Hf2 and MS-Hu6 (0.6 to 13 nM) for 4 hours followed by measurement of LUC activity using the Bright-Glo Luciferase Assay System (Promega, Catalog #E2610).

Preparation and Purification of Hu6-Fab

The Fab fragment of MS-Hu6 was prepared using a Pierce Fab Preparation Kit (ThermoFisher Scientific, Catalog #44985). Briefly, MS-Hu6 was digested with immobilized papain for 6 hours at 37°C. The digested sample was added to a Protein A column for 15 minutes to allow the binding of undigested and Fc regions of the digested antibody to the resin. The unbound Fab fragment (Hu6-Fab) was eluted using 0.1 M glycine.

X–Ray Crystallography

For crystallization, the Hu6-Fab protein (above) was purified further by passing through a Superdex 200 10/300 Increase Column (GE Healthcare) pre–equilibrated with 10 mM HEPES and 150 mM NaCl, at pH 7.5. Purity of the fractions was assessed by SDS-PAGE and the purest eluates were concentrated to 14.5 mg/mL. Protein was subjected to extensive crystallization trials using commercial screens with an automated crystallization robot (Douglas Instruments). Initial crystals were obtained from MCSG1 screen (Anatrace Products) using a sitting–drop vapor diffusion method at 20°C by mixing equal volumes of purified protein and crystallization conditions. Good quality, but very fragile plate–shaped crystals appeared in 3 weeks in a crystallization condition containing 0.1 M Tris-HCl (pH 8.5), 0.2 M ammonium sulphate, and 25% (w/v) polyethylene glycol 3350. These crystals were flash frozen without further cryoprotection. The best Hu6-Fab crystal diffracted X-rays to ~2.5 Å resolution with synchrotron radiation at

NECAT 24ID-C Beamline. The crystals belong to space group $P2_1$ with unit cell dimensions a=52.82 Å, b=50.08 Å, c=86.24 Å, α=γ=90°, and β=105.17°. The crystallographic asymmetric unit contains one molecule of each heavy and light chain in the unit cell. X–ray diffraction data measured at wavelength 0.9791 Å were indexed, integrated, and scaled using XDS, aimless, and various ccp4 suite programs (truncate, freeflag, and mtz2various) integrated into the RAPD pipeline at the NECAT 24ID Beamline.

Structure Determination and Refinement

To solve the structure with molecular replacement, search models for light and heavy chains were selected by running a BLAST program against PDB structures. For the light chain, 6OGX_D (with 97% Identity) and for the heavy chain, 5BMF_H (with 94% identity) were selected as 3D search models in Phaser. The resulting maps of the structure solution indicated clear electron densities for light and heavy chains (with top LLG of 744 and Top TFZ of 28). The structure solution was refined through phenix.refine for rigid–body, followed by positional and B–factor refinement with simulated annealing. Iterative model building was done in coot and the final refinement in phenix converged to Rwork=0.2135, Rfree=0.2630.

Computational docking

To model the interactions of Hu6-Fab with FSHβ, we used HADDOCK2.4 docking server (3). Coordinates of our experimentally determined Hu6-Fab crystal structure and previously determined FSH structure (PDB: 1XWD) were used for docking. Specifically, we utilized the well–characterized 13–residue–long epitope sequence from FSHβ (^{37}LVYKDPARPKIQK^{49}) and residues from all the CDRs from V_L and V_H domains for efficient docking. The lowest *Z*–score cluster of the complex was selected for analysis.

Dynamic Light Scattering

Colloidal stability of MS-Hu6 and Hf2 was evaluated using dynamic light scattering (DLS) (4). The method determines the size (hydrodynamic radius, rh) and homogeneity (polydispersity Index, PDI) of colloidal particles. In brief, 20 μL of formulated MS-Hu6 or Hf2 was diluted to 1 mL using either the formulation buffer or PBS. The resulting solution was then transferred to disposable 1 mL micro-cuvettes (Malvern, Catalog #ZEN0040). The diluted sample was examined using the Malvern Zetasizer Nano-ZS 90 system by subjecting it to a 90° scattering angle at 25°C for 60 seconds (5 cycles). The refractive index of the medium was fixed at 1.33, and dynamic viscosities were calculated. The results are presented as the *Z*–average of rh or PDI. All experiments were performed in duplicate, and representative particle size distribution (PSD) graphs are shown.

Pharmacodynamic Studies

To measure total, unbound and bound FSH levels, C57BL/6J mice (*N*=10) aged 8 to 12 weeks were ovariectomized, 1 week after which they were injected with vehicle or Hf2. Serum was collected weekly, up to 6 weeks. Total FSH was measured using ELISA (Sigma, Catalog #EEL097). To separate unbound and bound FSH fractions, protein A agarose beads (ThermoFisher Scientific, Catalog #20333) were pre–incubated with anti–mouse IgG Fc (ThermoFisher Scientific, Catalog #SA5-10275) overnight at 4ºC and washed with buffer (25 mM Tris-HCl, 150 mM NaCl; pH 7.2). The pre–coated beads were then incubated overnight at 4ºC with serum collected from Hf2–injected mice for the measurement of unbound FSH levels using the aforementioned ELISA kit. The beads were then washed and eluted with 0.1 M glycine at pH 2.5, and the eluate was used to measure the bound FSH levels using the same kit.

Measuring MS-Hu6 in Mouse Serum

The presence of humanized MS-Hu6 in mouse serum was detected using an in–house ELISA, wherein anti–human Fab (Sigma, Catalog #I5260) (100 μg) was used as a capturing antibody with overnight incubation at 4°C and anti–human Fc–conjugated with HRP (Sigma, Catalog #A0170) as the detection antibody.

Reproductive Parameters

To study the effect of Hf2 on the menstrual cycle, female C57BL/6J mice aged 10 to 11 weeks were injected with vehicle or Hf2 for 6 weeks (intraperitoneal, 200 μg, 3–days–a–week, *N*=6). To collect vaginal cells, 100 μL saline was gently introduced into the vagina using a pipette. Alter slow release, saline was drawn back into the tip, with the process repeated 4 to 5 times with the same sterile pipette tip. The procedure was repeated daily over 4 days of the estrus cycle to observe the proestrus, estrus, metestrus, and diestrus phases. Fluid from each draw, containing a few drops of cell suspension, was placed on a glass slide, air–dried, stained with 0.1% crystal violet (0.1 g crystal violet powder in 100 mL of de-ionized water), and viewed under a light microscope. We also sacrificed the mice to measure uterine weight by manual weighing. Serum estrogen levels were measured in groups of mice on a high–fat diet treated with vehicle or Hf2 by liquid chromatography/mass spectrometry (LC/MS) at the Brigham Research Assay Core (assay sensitivity 1 pg/mL).

Monkey Studies

Retired female African Green Monkeys (*Chlorocebus aethiops*), aged between 18 and 23 years, were utilized for safety studies at the Primate Center at Wake Forest School of Medicine. Monkeys were fasted overnight prior to sedation and anesthetized with ketamine injection (10–15 mg/kg). Sedated monkeys were maintained on a heated air blanket (Bair Hugger). Prior to each

injection, blood was collected (8 mL) for complete blood counts (CBCs) and serum chemistry and sent to IDEXX Laboratories. The first injection of MS-Hu6 was performed *via* intravenous infusion into the saphenous vein. Subcutaneous fluids (~100 mL) were administered during the initial injection, as supportive treatment for hydration and nutrition. All four monkeys were infused with 8 mg/kg of MS-Hu6, over a minute. Four further subcutaneous injections (8 mg/kg) were given into the lateral right thigh after every 4 weeks, during which time, body weight was recorded. To evaluate for acute safety, we recorded vital signs, namely heart rate, respiratory rate, arterial oxygen saturation (pulse oximetry) and rectal temperature after the first intravenous injection. Additionally, we monitored changes in skin color or appearance of a rash, as signs of anaphylaxis. All experiments and procedures were carried out on protocols approved by the Institutional Animal Care and Use Committee (IACUC) of Wake Forest School of Medicine.

<u>Diet–Induced Obesity</u>

To determine the minimal effective dose of MS-Hu6 required for the prevention of obesity, we have, as is customary for other humanized monoclonal antibodies (5-9), used the mouse analog of MS-Hu6, Hf2. We studied the effect of Hf2 on preventing diet–induced obesity in C57BL/6J mice on a high–fat diet *ad libitum* [Test Diet, 60% fat, Catalog #0056833 (58Y1)]. Groups of mice were injected with formulated Hf2 at different doses (10, 50, or 100 µg/day, 5 days–a–week) for 8 weeks or vehicle (formulation buffer). We measured the net food intake twice weekly and body weight weekly (using a digital weighing balance) and performed qNMR every week for 8 weeks. For the latter, mice were placed in a cylinder, which was placed in an EchoMRI-100H NMR analyzer (Echo Medical) to measure fat, lean and total mass, *per* manufacturer. At the end of the experiment, mice were sacrificed and all organs and fat depots were collected and weighed.

Novel Object Recognition Test

The Novel Object Recognition Test was performed in square test boxes (40 x 40 x 35 cm) with even lighting conditions (30 ± 5 lux). Each test box consisted of a grey steel bottom plate and white Perspex walls, with a camera mounted above all boxes. A tower of Lego bricks and a Falcon tissue culture flask filled with sand were used as objects (10). Prior to the experiments, both objects were tested with a separate cohort of mice to confirm that there was no object or side preference (data not shown). Placement of sample and novel objects followed a counterbalanced design between trials to control for order and location effects.

The test consisted of two trials, namely, sample and novel object trials, 24 hours apart. In the sample trial, mice were placed into the test box containing two equal sample objects (e.g., flasks) in front of the south wall facing away from the objects. Each mouse was allowed to explore the objects for 10 minutes before returning to its home cage. 24 hours later, the novel object trial was conducted by placing the mouse into the same test box containing one sample object and one unfamiliar object (e.g., a flask and a tower of Lego bricks) and object interaction was recorded for 10 minutes. After each trial, the objects and boxes were cleaned with a disinfectant (Quatricide, Pharmacal, Waterbury, CT) to eliminate odor cues. The test box was cleaned using 70% ethanol between each trial.

All test trials were video recorded, tracked, and analyzed with ANY-maze tracking software. Object interaction was defined as an event where the head of the mouse was within 2 cm of the object and directed towards the object, excluding sitting on the objects (11, 12). For the sample object trial, % object Interaction was calculated as (sample object interactions)/(total interactions) x 100%. For the novel object trial, % object interaction was calculated as (novel object interactions)/(total interactions) x 100% (13). Mice with <5% object interaction in either trial were excluded from the analysis (14).

Morris Water Maze Test

The Morris Water Maze Test was utilized to study acquisition and retrieval of consolidated memory[32]. For this, we use a circular pool (150 cm diameter) filled with water (26 ± 1°C; 10 cm distance from water surface to wall rim) that is made opaque with non–toxic tempera paint. A circular rescue platform (11 cm diameter; 27 cm from pool wall along the center line) is submerged 1–1.5 cm below the water surface and the testing area is illuminated with indirect lighting (150 ± 10 lux) to avoid reflections. To monitor mice during the trials, a camera is mounted to the ceiling centrally above the pool. The water maze is surrounded by black–and–white extra–maze cues on the walls of the room. Repeated episodes of excessive floating (>10 seconds/trial in ≥ 25% of trials) was found to be rare (6 mice during the entire study); the mice were excluded from the analysis.

For the spatial acquisition phase, a submerged rescue platform, invisible to the mice, is used. To locate the platform, mice use the extra–maze cues. While the platform location remains the same for all trials, the starting location varies between trials. Mice have 60 seconds to find the rescue platform. Each mouse performs four trials *per* day over 5–days with an inter–trial interval of 15 to 20 minutes. Mice that fail to locate the platform during the 60 second trial are placed on the platform for 15 seconds immediately after the end of the trial. Mean latency to reach the platform is calculated for each trial. For studying consolidated memory, we use a probe test in which mice are housed back in home cages on the 6th day for 24 hours without additional training. On day 7, the rescue platform is removed and the mouse is allowed to swim for 60 seconds. Percent of the time spent in the platform zone (40 cm diameter) surrounding the platform center point is determined.

Conjugation of MS-Hu6 to ^{89}Zr

MS-Hu6 (8.0 mg, 53 nmol) in PBS (0.93 mL) was basified using Na_2CO_3 buffer until a pH of 8.5 was reached. A solution of DFO-*p*-NCS in DMSO (0.12 mg, 160 nmol, 3.00 eq, 24 μL) was added gradually to the MS-Hu6 mixture in steps of ~5.0 μL. The resulting mixture was incubated at 37°C for 3 hours, manually agitated every 15 minutes, and then diluted with PBS (5.0 mL) and transferred to a 10 kDa MWCO centrifugal filtration tube (Vivaspin). The tube was spun at 4000 rpm until a volume of 0.5 mL remained. PBS (5.0 mL) was added and the mixture was again concentrated to a volume of 0.5 mL. This washing step was repeated twice. The DFO–functionalized MS-Hu6 was subsequently used for radiolabeling. For this, a solution of ^{89}Zr in oxalic acid (0.02 mL, 8.9 mCi) was diluted with PBS (0.15 mL) and neutralized using an aqueous Na_2CO_3 solution (1 M, ~16 μL) to reach a pH between 7.2 and 7.6. DFO–functionalized MS-Hu6 in PBS (0.5 mL) was added and the reaction was incubated for 40 minutes at 37 °C and 300 rpm using a thermomixer. Completion of ^{89}Zr chelation was confirmed by radio–TLC using aqueous EDTA (50 mM) as eluent. The mixture was purified using a PD10 size exclusion column using PBS as the eluent. Radiolabeled ^{89}Zr-MS-Hu6 was obtained with a radiochemical purity of >99% and a radiochemical yield of 80%.

Pharmacokinetics and biodistribution of ^{89}Zr-MS-Hu6

For pharmacokinetic studies, ^{89}Zr-MS-Hu6 was injected subcutaneously (250 μCi) into C57BL/6J mice (*N*=5, each, male and female). Blood (~5 μL) was collected *via* the tail vein at predetermined intervals: 0, 0.5, 1, 2, 4, 24, 48 and 72 hours, and the mice were sacrificed terminally. Blood was subjected to γ–counting (Wizard 2480 Automatic Gamma Counter, PerkinElmer). Values were corrected for decay and expressed as a percentage of the injected dose *per* gram of blood.

For biodistribution studies, ^{89}Zr-MS-Hu6 was injected subcutaneously (250 μCi) into C57BL/6J mice (*N*=3–5, male). Tissues of interest, namely kidney, liver, muscle, lung, heart, spleen, bone, bone marrow, brain, sWAT, vWAT, BAT, testis, adrenal gland, pancreas, and blood, were harvested at 24, 48, and 72 hours and γ counted. Values were corrected for decay and expressed as a percentage of the injected dose *per* gram of tissue.

References

1. Xiong J, Kang SS, Wang Z, Liu X, Kuo TC, Korkmaz F, Padilla A, Miyashita S, Chan P, Zhang Z, et al. FSH blockade improves cognition in mice with Alzheimer's disease. *Nature.* 2022;603(7901):470-476.
2. Galmozzi A, Sonne SB, Altshuler-Keylin S, Hasegawa Y, Shinoda K, Luijten IHN, Chang JW, Sharp LZ, Cravatt BF, Saez E, et al. ThermoMouse: an in vivo model to identify modulators of UCP1 expression in brown adipose tissue. *Cell Rep.* 2014;9(5):1584-1593.
3. van Zundert GCP, Rodrigues J, Trellet M, Schmitz C, Kastritis PL, Karaca E, Melquiond ASJ, van Dijk M, de Vries SJ, and Bonvin A. The HADDOCK2.2 Web Server: User-Friendly Integrative Modeling of Biomolecular Complexes. *J Mol Biol.* 2016;428(4):720-725.
4. Rojekar S, Pallapati AR, Gimenez-Roig J, Korkmaz F, Sultana F, Sant D, Haeck CM, Macdonald A, Kim SM, Rosen CJ, et al. Development and biophysical characterization of a humanized FSH-blocking monoclonal antibody therapeutic formulated at an ultra-high concentration. *Elife.* 2023;12:e88898.
5. Amanat F, Strohmeier S, Lee WH, Bangaru S, Ward AB, Coughlan L, and Krammer F. Murine Monoclonal Antibodies against the Receptor Binding Domain of SARS-CoV-2 Neutralize Authentic Wild-Type SARS-CoV-2 as Well as B.1.1.7 and B.1.351 Viruses and Protect In Vivo in a Mouse Model in a Neutralization-Dependent Manner. *mBio.* 2021;12(4):e0100221.
6. Clarke J, Leach W, Pippig S, Joshi A, Wu B, House R, and Beyer J. Evaluation of a surrogate antibody for preclinical safety testing of an anti-CD11a monoclonal antibody. *Regul Toxicol Pharmacol.* 2004;40(3):219-26.
7. Labrijn AF, Meesters JI, Bunce M, Armstrong AA, Somani S, Nesspor TC, Chiu ML, Altintas I, Verploegen S, Schuurman J, et al. Efficient Generation of Bispecific Murine Antibodies for Pre-Clinical Investigations in Syngeneic Rodent Models. *Sci Rep.* 2017;7(1):2476.
8. Schofield DJ, Percival-Alwyn J, Rytelewski M, Hood J, Rothstein R, Wetzel L, McGlinchey K, Adjei G, Watkins A, Machiesky L, et al. Activity of murine surrogate antibodies for durvalumab and tremelimumab lacking effector function and the ability to deplete regulatory T cells in mouse models of cancer. *MAbs.* 2021;13(1):1857100.
9. Thudium K, Selby M, Zorn JA, Rak G, Wang XT, Bunch RT, Hogan JM, Strop P, and Korman AJ. Preclinical Characterization of Relatlimab, a Human LAG-3-Blocking Antibody, Alone or in Combination with Nivolumab. *Cancer Immunol Res.* 2022;10(10):1175-1189.
10. Leger M, Quiedeville A, Bouet V, Haelewyn B, Boulouard M, Schumann-Bard P, and Freret T. Object recognition test in mice. *Nat Protoc.* 2013;8(12):2531-7.

11. Clark RE, and Martin SJ. Interrogating rodents regarding their object and spatial memory. *Curr Opin Neurobiol.* 2005;15(5):593-8.
12. Ennaceur A, and Delacour J. A new one-trial test for neurobiological studies of memory in rats. 1: Behavioral data. *Behav Brain Res.* 1988;31(1):47-59.
13. Bevins RA, and Besheer J. Object recognition in rats and mice: a one-trial non-matching-to-sample learning task to study 'recognition memory'. *Nat Protoc.* 2006;1(3):1306-11.
14. Taglialatela G, Hogan D, Zhang WR, and Dineley KT. Intermediate- and long-term recognition memory deficits in Tg2576 mice are reversed with acute calcineurin inhibition. *Behav Brain Res.* 2009;200(1):95-9.

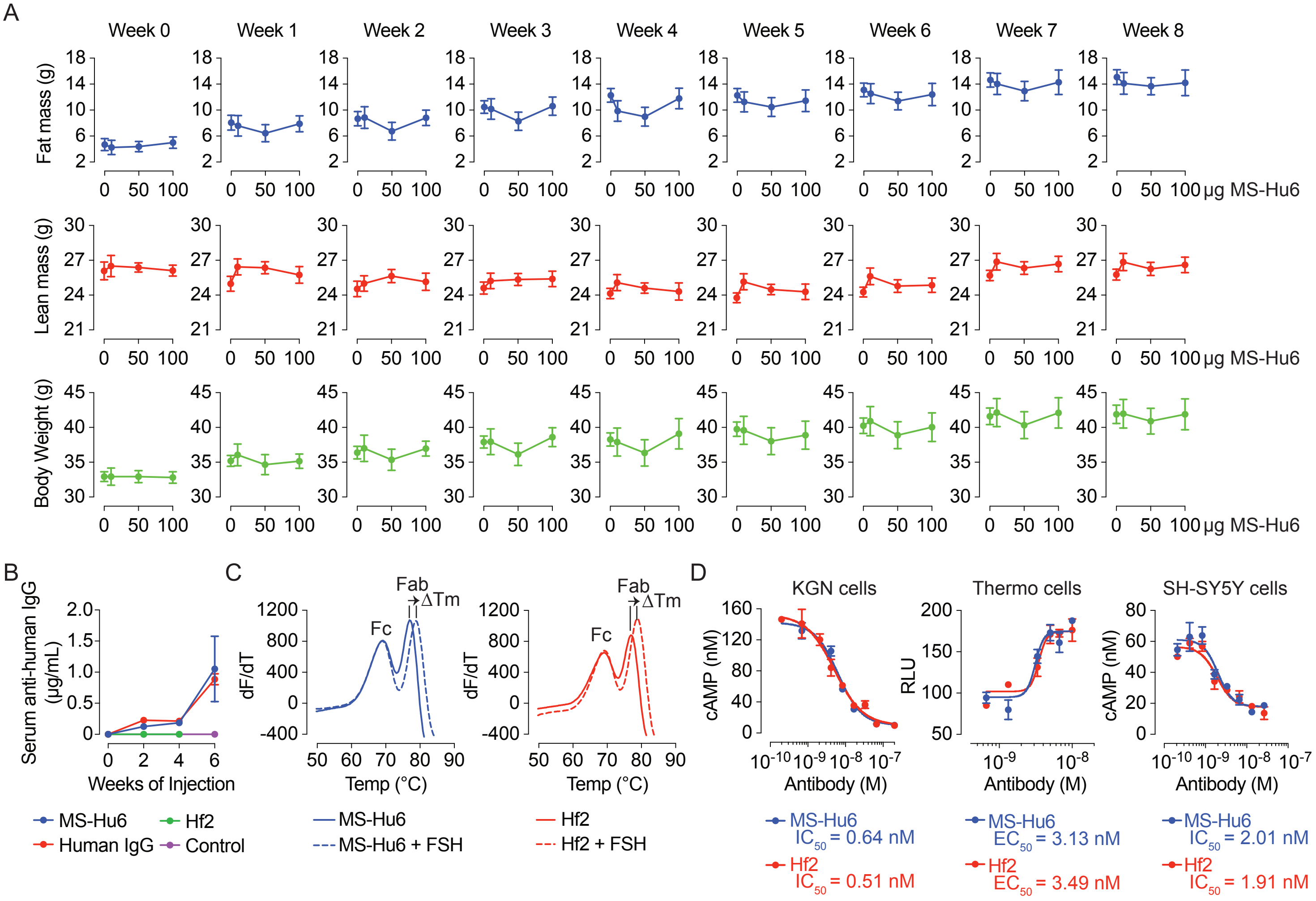

Figure S1: **Murine Hf2 as a Surrogate for Humanized MS-Hu6 for Long–Term *In Vivo* Studies in Mice.** Lack of effects of subcutaneously injected MS-Hu6 on fat mass, lean mass (qNMR) or body weight in female C57BL/6 mice on a high–fat diet up to 8 weeks (**A**). This was due to the induction in male C57BL/6 mice of anti–human IgGs (measured by an in–house ELISA) upon injection with human IgG or MS-Hu6, but not with Hf2 (*N*=7 mice *per* group) (**B**). Protein thermal shift assay, shown as melting curves (first derivative), showed that FSH binding to both MS-Hu6 and Hf2 shifted the melting temperature (T_m) of the Fab domain by ~1.8°C, and expectedly not of the Fc domain (**C**). There was also a concentration–dependent reduction of FSH–induced cAMP elevation with both Hf2 and MS-Hu6 in KGN human ovarian cancer cells, a concentration–dependent increase in luminescence (relative luminescence units, RLU) in dedifferentiated brown adipocytes (Thermo Cells) in which a *Ucp1* promoter is used to drive the expression of a luciferase reporter, and a concentration–dependent reduction in cAMP levels in SH-SY5Y neuroblastoma (FSH added at 5 µg/mL, 30 ng/mL and 5 µg/mL for ovarian cancer cells, adipocytes and neuroblastoma, respectively) (**D**). The data established equivalence between MS-Hu6 and Hf2 in terms of binding to and blocking the action of FSH. Statistics: *N*=10 mice per group for panel A; Mean ± SEM; two–tailed unpaired Student's *t*–test *vs*. vehicle.

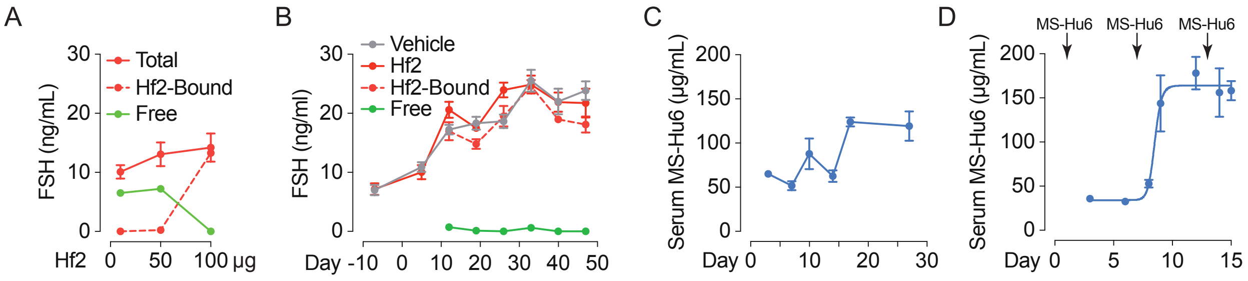

<u>Figure S2</u>: Pharmacodynamics and Pharmacokinetics of Hf2 and MS-Hu6 *In Vivo*. (**A**) Total, Hf2–bound and unbound (free) fractions of FSH in serum of C57BL/6 mice injected with Hf2, subcutaneously, 5 days a week for 8 weeks (*N*=10 mice per dose). (**B**) Female C57BL/6 mice were ovariectomized (*N*=20 mice). A week later, 10 mice were given vehicle, intraperitoneally, 5 days–a–week, with serum total FSH levels measured every week for 6 weeks. The other 10 mice were injected, in parallel, with Hf2 for the measurement of total FSH (5 mice), as well as bound and unbound FSH fractions (5 mice, each). Total FSH was measured using a commercial FSH ELISA kit, and bound and free FSH used an in–house ELISA (see 'Methods' for details). Detection of MS-Hu6 using an in–house ELISA (see 'Methods' for details) in serum of C57BL/6 male mice injected with MS-Hu6 (100 µg), subcutaneously, 5 days–a–week for 4 weeks (**C**) or intravenously at a cumulative dose of 500 µg per mouse per week (**D**), with serum sampling every 2 days (*N*=5 mice).

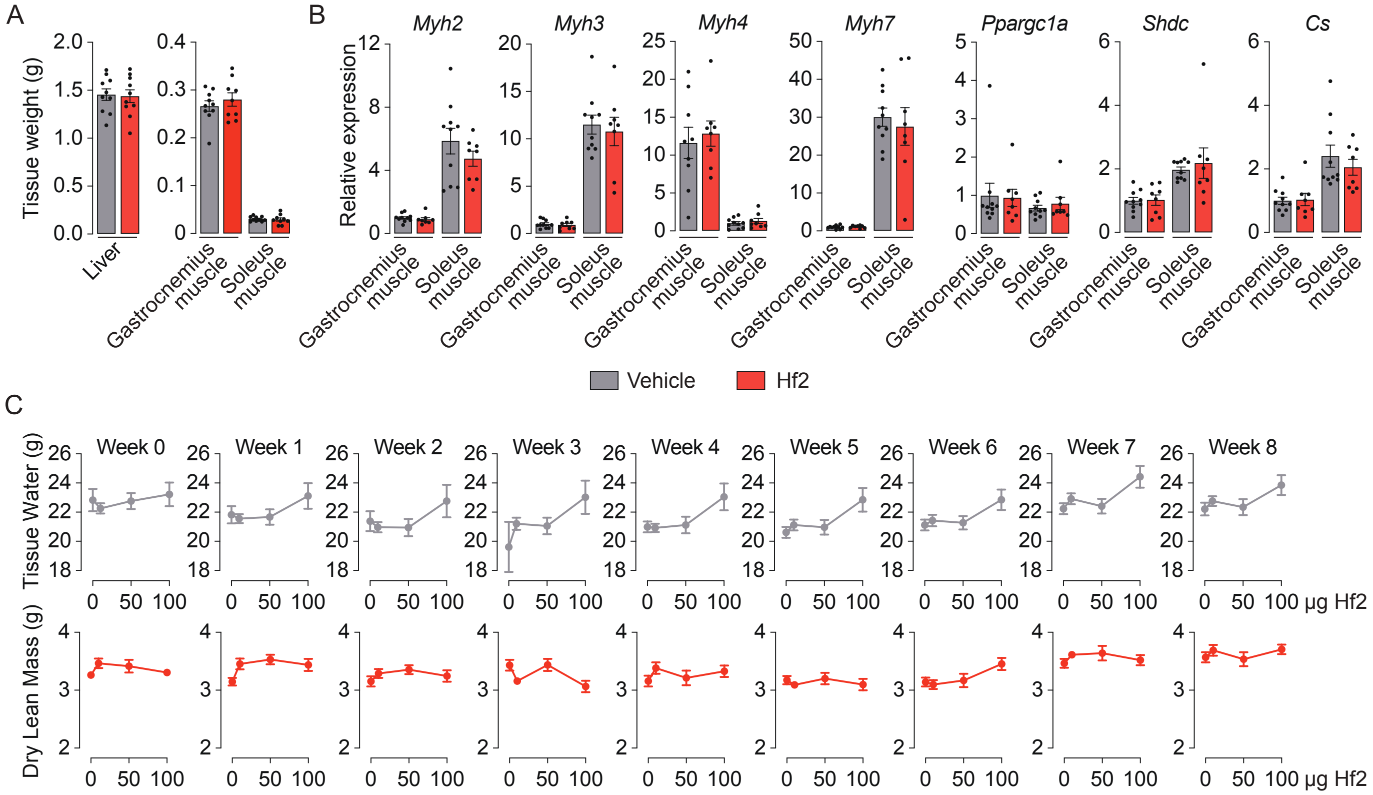

<u>Figure S3</u>: **Absent Effects of FSH Blockade on Lean Mass.** Groups of male C57BL/6 mice, matched for body weight and fed *ad libitum* on a high–fat diet, were injected with formulated Hf2 at different doses (10, 50, or 100 μg/day, 5 days–a–week) or formulation buffer for 8 weeks. No difference in weights of isolated liver, gastrocnemius muscle and soleus muscle between vehicle– and Hf2–treated mice (**A**). No differences in the expression of genes related to muscle strength, namely genes encoding myosin heavy chain (*Myh2*, *3*, *4* and *7*) and mitochondrial genes (*Ppargc1a*, *Sdhc*, and *Cs*) (**B**). Weekly quantitative nuclear magnetic resonance (qNMR) measurements showing the effect of formulated Hf2 on tissue water and dry lean mass (**C**). Statistics: Mean ± SEM (N=10); two-tailed unpaired Student's t–test.

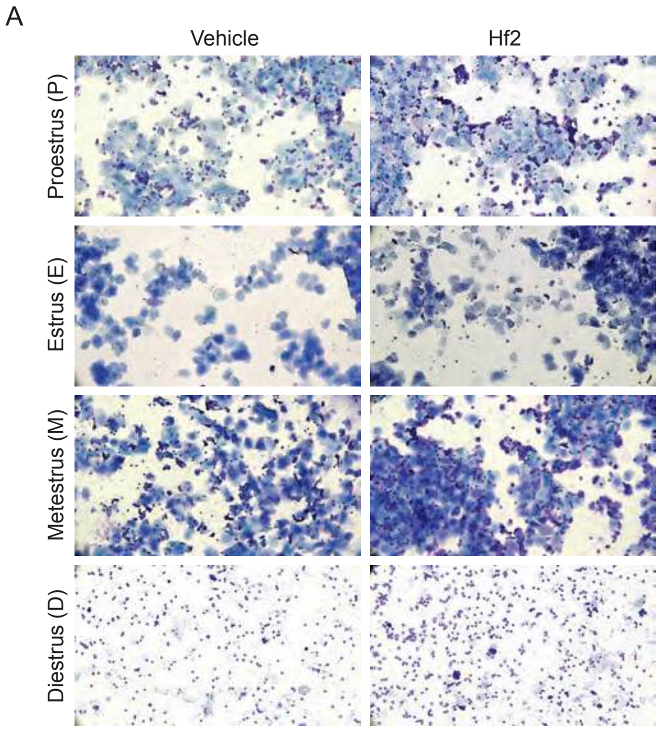

	Vehicle #1	Vehicle #2	Vehicle #3	Vehicle #4	Vehicle #5	Vehicle #6	Hf2 #1	Hf2 #2	Hf2 #3	Hf2 #4	Hf2 #5
Day 1	E-M	P	D	M-D	M	E	E-M	P	P	M-D	M
Day 2	M-D	E	D	D	M	M	E	E-M	P-E	P-E	P-E
Day 3	D	D	P	P	D	D	P	M-D	P-E	E	M-D
Day 4	E	M	E	E	D	P	M-D	D	M-D	M	D
# Phases	3	4	3	4	2	4	4	4	4	4	4

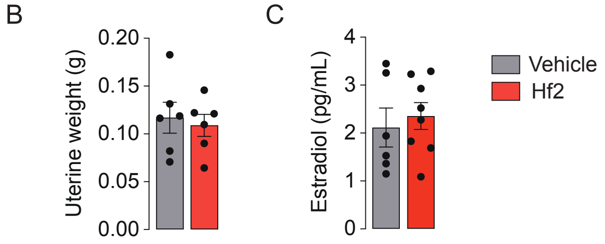

<u>Figure S4</u>: Absent Effects of FSH Blockade on Reproductive Functions. Groups of female C57BL/6 mice injected with Hf2 (intraperitoneal, 200 µg, 3–days–a–week) for 6 weeks. Representative images of crystal–violet–stained vaginal swabs showing no difference in morphology during proestrus (P), estrus (E), metestrus (M), or diestrus (D) between vehicle– and Hf2–treated groups (**A**). The number of menstrual cycle phases (A) or uterine weight (**B**) were also not different (*N*=6, 5 per group). In separate experiments, no difference in serum estrogen (LC/MS) in male C57BL/6 mice (on a high–fat diet) receiving Hf2 (100 µg/day, 5 days–a–week) or formulation buffer for 8 weeks (*N*=9 *per* group) (**C**). Statistics: Mean ± SEM; two-tailed unpaired Student's *t*–test.

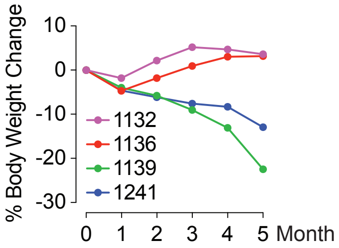

<u>Figure S5</u>: Body Weight of African Green Monkeys Treated With MS-Hu6. Individual body weights of retired 18– to 23–year old female monkeys (*N*=4; IDs: 1132, 1136, 1139, 1241) that were infused intravenously (IV) with MS-Hu6 (8 mg/kg), and subsequently given 4 further subcutaneous injections (8 mg/kg) 30 days apart.

Supplementary Table 1: Data collection and refinement statistics.

PDB ID	8VZW
Wavelength (Å)	0.9791
Resolution range	50.98-2.5 (2.59-2.5)
Space group	P 1 21 1
Unit cell	52.819 50.077 86.235 90 105.167 90
Total reflections	62209 (6312)
Unique reflections	15104 (1470)
Multiplicity	4.1 (4.3)
Completeness (%)	98.43 (99.12)
Mean I/sigma(I)	7.99 (2.07)
Wilson B-factor	39.08
R-merge	0.1448 (0.7321)
R-meas	0.1662 (0.8378)
R-pim	0.08012 (0.4008)
CC1/2	0.989 (0.834)
CC*	0.997 (0.954)
Reflections used in refinement	15044 (1467)
Reflections used for R-free	651 (82)
R-work	0.2135 (0.3292)
R-free	0.2630 (0.4041)
CC(work)	0.942 (0.860)
CC(free)	0.933 (0.712)
Number of non-hydrogen atoms	3260
macromolecules	3150
ligands	15
solvent	95
Protein residues	425
RMS(bonds)	0.006
RMS(angles)	0.86
Ramachandran favored (%)	96.39
Ramachandran allowed (%)	3.61
Ramachandran outliers (%)	0.00
Rotamer outliers (%)	3.43
Clashscore	6.61
Average B-factor	41.97
macromolecules	41.95
ligands	48.91
solvent	41.52

*Statistics for the highest-resolution shell are shown in parentheses.

##	Hu6-Fab	FSH	Dist. [Å]
1	L:TYR50[OH]	B:ARG35[NH1]	2.93
2	H:ASP32[OD2]	B:LYS40[NZ]	2.55
3	H:THR97[N]	B:ASP41[OD2]	2.65
4	H:THR97[OG1]	B:ASP41[OD2]	2.70
5	H:GLY98[N]	B:ASP41[OD2]	3.26
6	H:TYR33[OH]	B:ASP41[OD1]	2.90
7	H:TYR33[OH]	B:ALA43[N]	2.94
8	L:TYR32[OH]	B:ARG44[NH1]	3.09
9	L:ARG53[NH1]	B:PRO45[O]	2.76
10	H:SER54[OG]	A:GLY73[O]	3.20
11	H:SER31[O]	A:LYS75[NZ]	3.00
12	H:SER53[OG]	A:LYS75[NZ]	2.81
13	H:ASP32[OD2]	A:LYS75[NZ]	3.83
14	H:ASP32[OD1]	A:LYS75[NZ]	3.10

Supplementary Table 2: Hydrogen bonding at Hu6-Fab:FSH complex interface.
